# CUL5-mediated ubiquitination in cancer cell therapy: context-dependent roles, molecular networks, and emerging therapeutic avenues

**DOI:** 10.3389/fmed.2026.1775447

**Published:** 2026-04-17

**Authors:** Yuanchen Lu, Yichen Qian, Yue Ma, Xianhua Shao, Jianjun Xie

**Affiliations:** Department of Urology, Suzhou Municipal Hospital, Gusu School, The Affiliated Suzhou Hospital of Nanjing Medical University, Nanjing Medical University, Nanjing, China

**Keywords:** CUL5, malignant tumor, neddylation, targeted therapy, tumor cell therapy, ubiquitination

## Abstract

As the core scaffold protein of the Cullin-RING ligase 5 (CRL5) complex, CUL5 regulates the stability of multiple substrate proteins through the ubiquitin-proteasome system (UPS), playing a crucial role in the initiation, progression, and cellular therapy of malignant tumors. This review systematically elaborates the context-dependent role, molecular regulatory network, and therapeutic targeting potential of CUL5-mediated ubiquitination in cancer cell therapy. The activity of CUL5 is highly dependent on NEDD8-mediated neddylation, and its dysregulation indirectly influences tumor cell proliferation, apoptosis, metabolic reprogramming, angiogenesis, and the immune microenvironment by modulating key signaling pathways such as NOXA, mTORC, TRAF6/NF-κB, and JAK/STAT. Notably, CUL5 exhibits dual regulatory functions in various cancers, and its expression level correlates differently with prognosis depending on tumor type. In recent years, the development of inhibitors and nano-delivery systems targeting CUL5 and its related pathways has provided novel strategies for precisely targeting CUL5. Moreover, in adoptive cell therapies (e.g., CAR-T, TCR-T, CAR-NK), modulation of CUL5 expression can significantly enhance immune-cell proliferation, cytokine secretion, and anti-tumor efficacy. This article summarizes the multidimensional role of CUL5 in tumor cell therapy and prospects its potential as a novel therapeutic target in combined therapies and precision medicine.

## 1 Background

The ubiquitin-proteasome system (UPS) consists of the E1 ubiquitin-activating enzyme, E2 ubiquitin-conjugating enzyme, and E3 ubiquitin ligase (1). Among these components, E3 ubiquitin ligases play a decisive role in determining substrate specificity. Based on structural motifs, E3 ubiquitin ligases are broadly categorized into three families: homologous to the E6-AP carboxyl terminus (HECT), really interesting new gene zinc finger domains (RING), and RING-between-RING ligases (RBRL) (2). RING constitute approximately 90% of known E3 ligases, with Cullin-RING Ligases (CRLs) representing the most prevalent subclass responsible for catalyzing the ubiquitination of approximately 20% of cellular proteins (3–5).

CUL5, also known as VACM-1, was initially cloned from a rabbit renal medullary cDNA library and identified as an antidiuretic hormone receptor-associated protein that exhibits upregulation under osmotic stress conditions (6–8). Subsequent studies established its identity as a member of the cullin family (9), with its encoding gene localized to chromosome 11q22-23 (7). Functional investigations have demonstrated that CUL5 modulates the stability of multiple downstream substrates through ubiquitin-mediated proteolysis (10).

CUL5 expression is frequently downregulated in various malignancies—including breast cancer (11), renal cancer(12) and endometrial cancer (13)—and is associated with poor patient prognosis (14). Paradoxically, elevated CUL5 levels have been observed in certain cancers, such as colorectal cancer (15) and hepatitis B virus-induced hepatocellular carcinoma (16), and are correlated with adverse outcomes in adrenocortical carcinoma (14). These findings underscore the highly context-dependent regulatory roles of CUL5 in cancer progression. The molecular mechanisms underlying this functional duality warrant further investigation.

The direct and precise targeting of CUL5 for therapeutic purposes poses significant challenges due to its broad physiological functions and context-dependent effects. Currently, few drugs directly target CUL5; however, recent developments in inhibitors targeting the CUL5-related pathways (17, 18) and nano-delivery systems (19) have provided novel strategies for its precise targeting. Furthermore, in adoptive cell therapies such as CAR-T, TCR-T, and CAR-NK (20–22), modulating CUL5 expression has been shown to significantly enhance immune cell proliferation, cytokine secretion, and anti-tumor efficacy. Thus, CUL5 still holds considerable potential as a key target in cancer cell therapy, which is broadly defined as therapeutic strategies that directly target or utilize cells—including tumor cells and immune cells—for cancer treatment (23).

In light of the above, this narrative review systematically synthesizes recent advances in the mechanistic roles of CUL5 in malignant tumors and its targeted therapeutic strategies, with a particular focus on cancer cell therapy. A comprehensive literature search was performed using PubMed and Web of Science, prioritizing peer-reviewed original research and high-impact reviews published within the last 5 years (2021–2025). Seminal early studies offering critical mechanistic insights were also included, and additional relevant articles were identified through reference mining. This review aims to provide investigators in the field with comprehensive and cutting-edge theoretical insights and research directions.

## 2 Structure of the CRLs family

**FIGURE 1:**
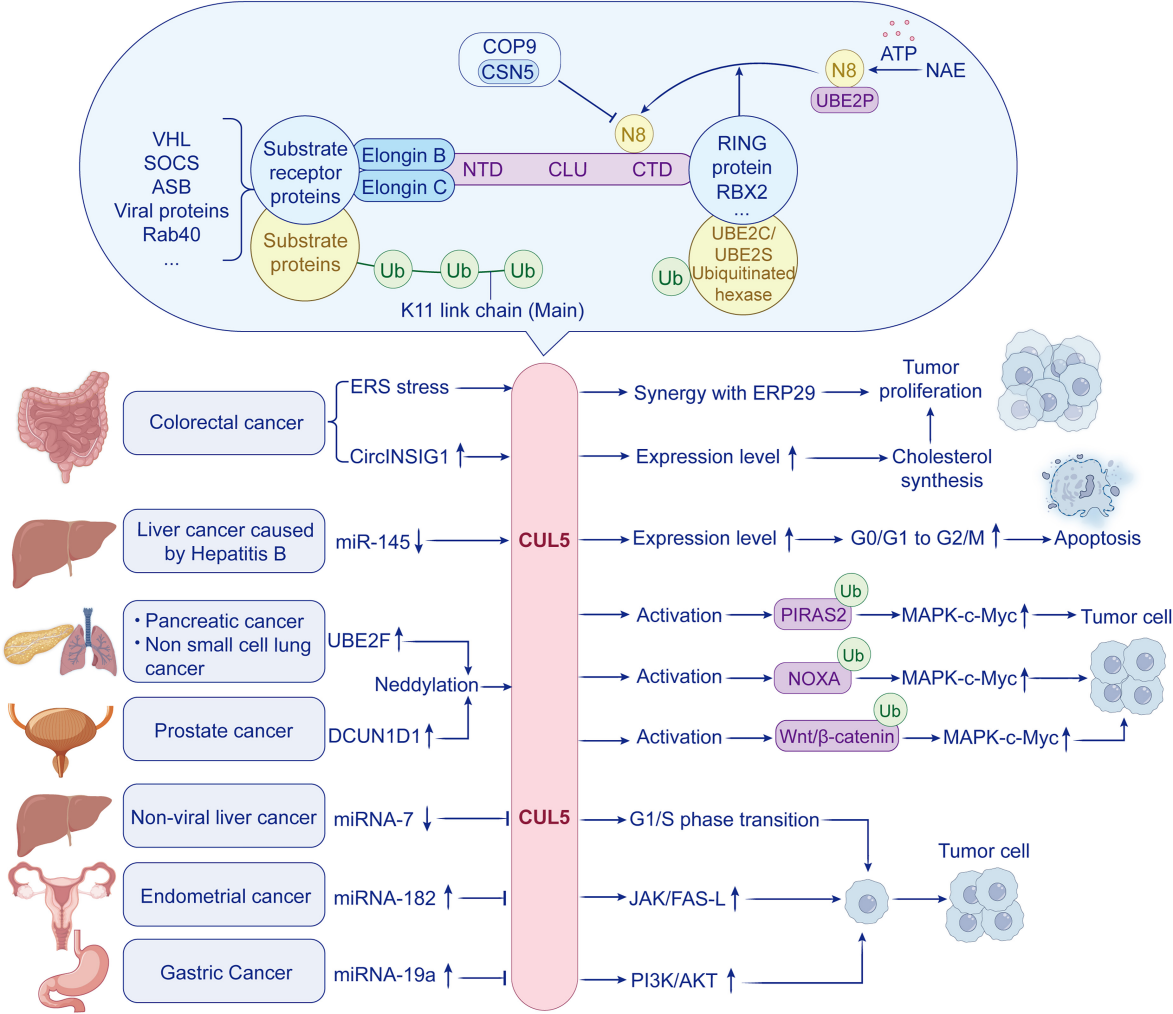
Structure, function, and regulatory mechanisms of CRL5, along with the upstream and downstream signaling pathways of CUL5 in different tumors.

Cullin-RING Ligases (CRLs) are multi-subunit complexes composed of substrate receptor proteins, adaptor proteins, a catalytic subunit (RING protein), and a Cullin protein. Substrate receptor proteins, such as von Hippel-Lindau tumor suppressor (VHL), suppressor of cytokine signaling (SOCS) (24), the ASB-box protein family (25), iral proteins (e.g., Vif, viral infectivity factor) (26, 27), F-box (28), and Bric-a-brac/Tramtrack/Broad-complex (BTB) domains (29), are responsible for directly recognizing substrates and determining enzymatic specificity (20, 30). These receptors contain specialized substrate-binding modules (SBMs) that interact with the N-terminal domain of cullin proteins (31). For instance, VHL recruits Elongin C, Elongin B, Cul2, and Rbx1 to form functional complexes, whereas SOCS family members assemble with Elongin C, Elongin B, CUL5, and Rbx2 (32). Adapter proteins, notably Elongin B/C heterodimers, mediate physical linkage between cullin scaffold proteins and substrate receptors. The catalytic RING subunits, RBX1 (RING-box protein 1) and RBX2 (also termed SAG/ROC2/RNF7), facilitate ubiquitin transfer by interacting with E2 ubiquitin-conjugating enzymes. This interaction enables direct ubiquitination of targeted substrates through a thioester intermediate on the E2 enzyme (Figure 1) (33, 34). Cullin proteins serve as the scaffold components of CRLs (35). Their gene family is evolutionarily conserved, and members within the family can influence one another to modulate the ubiquitination levels of substrates (35). For example, the CRL4 complex mediates neddylation of Elongin C via recognition of specific degradation signals (degrons), which leads to reduced activity of CRL5 complexes and subsequent attenuation of substrate ubiquitination (36). Additionally, studies by Zhou et al. (37) demonstrated that stress-induced activation of CUL3 results in functional inhibition of CUL5, highlighting the intricate regulatory networks within cullin-dependent ubiquitination systems.

## 3 A unifying framework: the determinants of CUL5’s context-dependent functions

As the scaffold protein of the CRL5 complex (35), CUL5 intrinsically lacks substrate specificity. Whether it ultimately functions as a tumor promoter or suppressor (38) is not determined by CUL5 itself, but rather by distinct substrate receptors recruited in specific cellular contexts (35). The following factors may modulate CRL5 functionality and influence CUL5’s selection of substrate receptors and substrates: (1). Cell type and state: is it a tumor cell, immune cell, or other cell type? Is the cell in an activated state (21)? (2). Molecular subtyping and genetic background of tumors: different expression profiles across cancers (13) affect which substrate receptors bind to CUL5. (3). Tumor microenvironmental signals: signals such as hypoxia (39), cytokines (e.g., IFN-γ) (12), and stress (40) can regulate the expression, modification, and localization of CUL5 or its receptors, thereby dynamically altering CRL5 complex composition and activity. (4). Post-translational modification status: neddylation of CUL5 serves as a critical activation switch (41, 42), and dysregulation of this process represents an upstream event determining its functional trajectory.

### 3.1 SOCS and CUL5

The SOCS protein family comprises eight members, which bind to Elongin B/C and recruit CUL5 to form CRL5 complexes. Among these, SOCS-1 and SOCS-3 exhibit the weakest binding affinity (43–45). The C-terminal region of the SOCS-box domain, termed the Cul5 box, is implicated in mediating specific interactions between SOCS-box proteins and the CUL5-RBX2 catalytic module (34).

Inhibition or reduced expression of CUL5 binding with SOCS proteins leads to various abnormalities. For instance, in small cell lung cancer (SCLC), loss of SOCS3/CUL5 leads to stabilization of integrin β1, an extracellular matrix receptor. This prevents its degradation, enhances cell-matrix adhesion, and activates focal adhesion kinase (FAK)/SRC signaling pathways, thereby driving tumor metastasis (46). In pancreatic cancer, decreased formation of the CUL5-SOCS6 complex promotes tumor cell survival and proliferation (44). Furthermore, disruption of CRL5 activity results in hippocampal dentate gyrus morphological abnormalities, disorganized neuronal alignment, and reduced numbers/maturity of newborn neurons, culminating in cognitive impairments in murine models (47). Mechanistically, this phenotype may involve SOCS-dependent pathways. Mechanistically, Simó et al. (48) demonstrated that the SOCS7-CRL5 complex ubiquitinates phosphorylated Dab1, promoting its degradation and suppressing Reelin signaling hyperactivation. This regulatory mechanism terminates neuronal migration by lowering phosphorylated Dab1 levels. Similarly, SOCS4-CUL5 complexes ubiquitinate tau in human neurons, modulating its stability and correlating with susceptibility to tauopathies in both mice and humans (49). In summary, the SOCS family serves as an integral functional component of CUL5, and loss of its function promotes tumor metastasis, neurodevelopmental abnormalities, and cognitive impairment.

### 3.2 Viral proteins as substrate receptors: hijacking CUL5 for immune evasion and tumorigenesis

To evade host immune surveillance or facilitate their own replication, numerous viruses have evolved proteins capable of hijacking the host CRL5 system. The Viral Infectivity Factor (Vif) of Human Immunodeficiency Virus-1 (HIV-1) acts as a virally encoded substrate receptor protein. Its zinc-binding region directly interacts with Cul5, specifically targeting the host antiviral restriction factor APOBEC3G (A3G) for ubiquitination and degradation. This reduces the editing of viral DNA by APOBEC3G, thereby enhancing the replicative capacity and immune evasion of HIV-1 (26, 27). Similarly, proteins such as E4orf6 and E1B55K from Adenovirus (50), the LANA protein from KSHV (Kaposi’s sarcoma-associated herpesvirus) (51), and the BZLF1 protein from EBV (52, 53) can promote the ubiquitination of the antitumor factor p53 and other tumor suppressor proteins via CUL5. This ultimately leads to genomic instability, cellular transformation, and tumorigenesis (54). Thus, inhibiting CUL5-E3 ligase activity may enhance the stability of tumor suppressor proteins.

Cyclic GMP-AMP synthase (cGAS) is a cytosolic DNA sensor that activates innate immunity and is crucial for cellular senescence (55). However, within the nucleus, cGAS exacerbates genomic instability by inhibiting homologous recombination repair (HR), thereby promoting tumorigenesis (56). Xu et al. (57) discovered that CRL5 (Cullin-RING Ligase 5) forms a complex with SPSB3 (SPRY domain and SOCS box-containing protein 3). This complex recognizes nuclear cGAS via SPSB3 and promotes its ubiquitination and degradation. Accumulation of nuclear cGAS activates the type I interferon signaling pathway, leading to enhanced innate immune responses. Leveraging this mechanism, they found that disrupting SPSB3-mediated cGAS degradation increases cellular sensitivity to type I interferon signals, thereby improving resistance to DNA viruses such as HSV-1 and VACV. Consequently, this mechanism could potentially be exploited in tumors to regulate CRL5-mediated cGAS degradation and modulate anti-tumor immunity.

### 3.3 Small GTPases as substrate receptors for CUL5

Small GTPases, members of the Ras superfamily, regulate their activity through binding to GTP (guanosine triphosphate) or GDP (guanosine diphosphate), playing pivotal roles in intracellular signaling (58).

Small GTPases, members of the Ras superfamily, regulate their activity through binding to GTP (guanosine triphosphate) or GDP (guanosine diphosphate) and play essential roles in intracellular signal transduction. Rab40 is an atypical Rab protein characterized by a C-terminal SOCS box motif. As a substrate receptor, Rab40 assembles with CRL5 to form an active E3 ubiquitin ligase complex (59, 60) and ubiquitinates AMBRA1. Notably, this modification does not lead to AMBRA1 degradation but instead appears to induce AMBRA1-dependent transcriptional regulation, thereby modulating cell adhesion and migration (61). Rab40b binds to Cul5 to form a functional CRL5 complex that ubiquitinates Rap2, promoting its recycling from endosomal/lysosomal compartments to the leading edge of the plasma membrane, where it interacts with actin filaments to drive cell migration (60). Duncan et al. (62) demonstrated that Rab40b also plays a critical role in breast cancer cell migration. Upon forming the CRL5 complex, it is hypothesized to ubiquitinate and degrade negative regulators of matrix metalloproteinases (MMP2/9), thereby enhancing their secretion and subsequently promoting cell migration and invasion.

Rab40c interacts with the CUL5-elongin B/C complex to ubiquitinate and degrade ankyrin repeat-containing protein 28 (ANKRD28), a scaffold subunit of the PP6 phosphatase complex. By inhibiting PP6-mediated dephosphorylation, this degradation activates focal adhesion kinase (FAK) and MOB1, reducing focal adhesion density and size while enhancing migratory capacity (63). Beyond tumor biology, CRL5 regulates neural stem cell differentiation and neuronal maturation by maintaining proteostasis, ensuring normal cognitive function and behavior (64). In cortical development, CUL5 functions as a key regulator of hippocampal morphogenesis by ubiquitinating small GTPases ARL4C and ARF6. Reduced CUL5 expression diminishes ubiquitination of Dab1, a Reelin signaling effector and cytoplasmic adaptor protein, leading to excessive neuronal migration and disrupted cortical layering (65).

In summary, small GTPases—particularly the Rab40 family—function as substrate receptors that assemble with CUL5 to form ubiquitin ligase complexes, thereby regulating cell migration through ubiquitination and degradation of diverse substrate proteins.

### 3.4 CUL5 and neddylation

The covalent attachment of NEDD8 to Lys724 at the C-terminus of CUL5—termed neddylation—induces conformational changes that activate CRL5 (41, 42). This modification is catalyzed by an enzymatic cascade consisting sequentially of the NEDD8-activating enzyme E1, NEDD8-conjugating enzyme E2, and substrate-specific NEDD8-E3 ligase (66). Two E2 enzymes, UBE2M and UBE2F, exist (67), with the latter exhibiting relative specificity for binding RBX2/SAG to promote Cul-5 neddylation (68). Three primary E3 ligases are involved: RBX1 (RING-box protein 1), RBX2 [RING-box protein 2; also known as SAG (sensitive to apoptosis gene)/ROC2 (regulator of cullins 2)/RNF7 (ring finger protein 7)], and DCN1 (69, 70).

Cullin and RBX proteins form a “C/R” domain through an intermolecular β-sheet—a structure that arises from the co-folding of the cullin α/β domain and the N-terminal region of RBX upon binding (28). Notably, RBX2/SAG functions dually as both the E3 ligase for Cul5 neddylation and the E3 ligase for CRL5 ubiquitination (41). Specifically, RBX2 binds Cul5 via its N-terminus and engages ubiquitination E2 enzymes (UBE2C and UBE2S) through its RING domain, thereby acting as a ubiquitination E3 ligase that catalyzes the transfer of ubiquitin from the E2 to substrates, predominantly assembling polyubiquitin chains via K11 linkages This interaction enables direct ubiquitination of targeted substrates through a thioester intermediate on the E2 enzyme (33, 34, 41); however, K48-linked ubiquitination has also been reported (Figure 1) (71, 72). E2 enzymes serve as the catalytic units that execute ubiquitin chain elongation and, to a large extent, determine linkage specificity (73). UBE2S has been demonstrated to exclusively synthesize K11 linkages, while UBE2C also primarily generates K11 chains (74). In the context of the master cell cycle regulator APC/C, UBE2C initiates the formation of K11, K48, and K63 linkages, whereas UBE2S specifically assembles K11-branched structures, giving rise to K11/K48-branched ubiquitin chains (75). These findings collectively provide a mechanistic rationale for the linkage preference observed in CRL5-mediated ubiquitination.

Cullin 1–4 predominantly form stable binary complexes with RBX1 (76), and the key residues within their WHB (Winged Helix B) domains that mediate non-covalent NEDD8 binding are evolutionarily conserved (77). In contrast, RBX2 primarily associates with CUL5 (78, 79), and the WHB domain of CUL5 adopts a distinct conformation upon NEDD8 engagement. NEDD8 modification induces conformational remodeling of the CUL5–RBX2 complex, alleviating its autoinhibited state and exposing an interface competent for binding the E2∼ubiquitin (E2∼Ub) conjugate. This conformational change positions the RBX2–E2∼Ub module in closer spatial proximity to the substrate protein recruited by the N-terminal domain of CRL5 (80), thereby shortening the effective distance between the E2 active site and the substrate lysine residue. This markedly enhances the efficiency of ubiquitin transfer and facilitates both RBX2–E2 approximation and substrate ubiquitination (81).

The detailed mechanisms of neddylation are continuously being elucidated. For instance, the availability of crystal structures for both the unmodified and neddylated CUL5–RBX1 complex allowed Onel et al. (82) to directly compare conformational changes induced by neddylation. They observed that NEDD8 attachment to the H29 region of CUL5 induces a transition from a closed to an open state, regulating the rotation of the Rbx1 RING domain via a dynamic hinge mechanism, thereby repositioning Rbx1 relative to the E2 enzyme. Given the high structural conservation between CUL5’s binding modes with RBX1 and RBX2 (34), the allosteric regulatory mechanism of neddylation revealed by the CUL5–RBX1 model is likely applicable to the CUL5–RBX2 complex as well. Furthermore, Sebastian et al. (83) found that neddylation-induced structural rearrangement of CUL5 facilitates its interaction with another key RBR-type E3 ligase, ARIH2, thereby activating the ubiquitin ligase activity of CUL5. This activated CUL5–ARIH2 complex promotes substrate ubiquitination, although the downstream pathways regulated by this mechanism remain to be fully elucidated.

#### 3.4.1 Neddylation and cancer

The functional activity of CUL5 is critically dependent on NEDD8 modification (neddylation) (Figure 1); however, its hyperactivation has been implicated in disease progression and poor prognosis across multiple human cancers. Vava et al. (84) demonstrated that overexpression of DCUN1D1 in prostate cancer enhances NEDD8 transfer to cullin proteins (85), promoting neddylation of CUL5 and other cullin family members. This post-translational modification activates the Wnt/β-catenin signaling pathway, thereby driving prostate cancer cell proliferation. Given its upregulation in various malignancies, DCUN1D1 likely exerts oncogenic effects by modulating cullin neddylation and downstream signaling pathways (86). In pancreatic cancer, UBE2F forms complexes with CRL5 to ubiquitinate and degrade DIRAS2, a tumor suppressor protein. This degradation activates the MAPK-c-Myc signaling axis, resulting in enhanced cancer cell proliferation and tumor growth (87). These findings suggest that inhibiting CUL5 neddylation may represent a promising therapeutic strategy for cancer treatment. Sundquist et al. (88) reported that mutation of the neddylation-critical lysine residue Lys724 in CUL5 disrupts its ubiquitin ligase activity, leading to suppression of proliferation-related signaling pathways and reduced cell growth. Furthermore, PKA-mediated phosphorylation of serine 730 (S730) residue in CUL5 enhances its neddylation efficiency and nuclear localization, potentiating its anti-proliferative effects (9).

#### 3.4.2 The NEDD8/CUL5/NOXA axis

NOXA (Phorbol-12-myristate-13-acetate-induced protein 1), a pro-apoptotic protein (89), modulates cell death by influencing the ubiquitination and degradation of itself through CRL5, which is regulated by key molecules in the CUL5 neddylation process. In colorectal cancer (CRC), Xu et al. (90) demonstrated that PRDX1 forms oligomers in response to elevated intracellular reactive oxygen species (ROS) levels. These oligomers associate with UBE2F and CUL5 to assemble a ternary complex, which enhances CUL5 neddylation. The activated CRL5 complex subsequently ubiquitinates NOXA via K11-linked polyubiquitin chains, leading to NOXA proteasomal degradation and subsequent suppression of CRC cell apoptosis. Similarly, Zhou et al. (91) confirmed that UBE2F-activated CRL5 promotes NOXA degradation through K11-linked (rather than K48-linked) ubiquitination, thereby enhancing survival of lung cancer cells. To counteract this effect, Xu et al. (92) employed HA-9104, a selective inhibitor that binds UBE2F and blocks CUL5 neddylation. This intervention led to CRL5 inactivation across multiple lung cancer cell lines, resulting in NOXA accumulation, induction of apoptosis, and increased radiosensitivity. These findings highlight the therapeutic potential of developing highly specific inhibitors targeting key molecules in the CUL5 neddylation pathway, as such agents could selectively suppress CRL5 activity and delay NOXA degradation.

Under stress conditions, UBE2M is induced and functions as a dual E2 enzyme. It not only participates in the neddylation of CUL-3, but also associates with the Parkin/DJ-1 E3 complex to promote the degradation of UBE2F. This leads to the inactivation of CUL-5, resulting in the accumulation of its substrate NOXA, which subsequently induces apoptosis and suppresses tumor growth (37). This study establishes a novel cross-talk network between E2 and E3 enzymes.

Numerous studies have explored strategies to regulate CRL5 activity by targeting key molecules in the neddylation pathway. Yue Ma et al. (93) synthesized WS-299, which binds to RBX1 in gastric cancer cells and disrupts the interaction between RBX1 and UBE2M. This selectively inhibits the neddylation of CUL3/5, leading to significant accumulation of the CRL3 and CRL5 substrates Nrf2 and NOXA. The accumulation of NOXA promotes tumor cell apoptosis by inhibiting anti-apoptotic BCL-2 family proteins such as MCL-1. However, the concurrent accumulation of Nrf2 antagonizes the pro-apoptotic effect of NOXA. Therefore, combining WS-299 with an Nrf2 inhibitor (e.g., siNrf2) synergistically enhances ROS levels, increases apoptotic cell numbers, and suppresses tumor growth *in vivo*. Additionally, Soucy et al. (3) discovered a small-molecule inhibitor named MLN4924, which selectively inhibits the NEDD8-activating enzyme (NAE) and thereby blocks the initial step of the neddylation pathway. It non-specifically inhibits the activity of Cullin-RING ligases (CRLs), leading to the accumulation of CRL substrates such as p27 and NOXA, ultimately inducing apoptosis (94). However, due to its non-selective suppression of the neddylation pathway, MLN4924 also promotes the accumulation of pro-tumorigenic factors such as PD-L1 (95), which can enhance cancer cell proliferation and immune evasion to some extent (96). These findings underscore both the promise and the challenges of targeting the neddylation pathway for cancer therapy, as well as the need for selective neddylation inhibitors to minimize off-target effects while maximizing therapeutic efficacy.

#### 3.4.3 The COP9 signalosome (CSN) and CUL5

The COP9 signalosome (CSN), an eight-subunit protein complex, regulates cullin-RING ligase (CRL) activity through its Jab1/MPN metalloenzyme-like domain, which catalyzes NEDD8 hydrolysis to return CRLs—including CRL5—to their inactive closed conformation, a process termed deneddylation (Figure 1) (82, 97). The dynamic equilibrium between neddylation and deneddylation is critical for maintaining functional homeostasis in CRL complexes (98).

Cope et al. (99) identified COPS5 (CSN5) as a subunit of the CSN complex, which is capable of removing Nedd8 from Cul1, thereby regulating Cul1 activity. It has been established that CSN5 serves as the catalytic core of the CSN complex, and the rate of deneddylation for all CUL proteins depends on their binding affinity to CSN5 (100). It is hypothesized that CUL5 may also be regulated by CSN5. This was confirmed by Zhang et al. (101), whose study demonstrated that TNF-α activates the IKK complex (IKKα and IKKβ), thereby initiating the NF-κB signaling pathway. This activation also phosphorylates serine 201 (S201) and threonine 205 (T205) residues on CSN5, reducing the CSN complex’s deneddylation capacity. This prolongs CRL5 activity, leading to the ubiquitination and degradation of IκB (an inhibitor of NF-κB). Consequently, NF-κB translocates to the nucleus, triggering the transcription of target genes that regulate cell survival and immune responses. These findings highlight the therapeutic potential of targeting CSN5, the catalytic hub of the CSN complex, to indirectly modulate CRL5 activity.

## 4 CUL5 regulates genetic material

The regulation of genetic material synthesis and repair by CUL5 propels malignant tumor progression. PAICS (phosphoribosylamine-glycine ligase), a key enzyme in de novo purine synthesis (DNPS), is essential for cell proliferation and DNA synthesis (102). Under cellular stress, the expression of ASB11 is upregulated. ASB11 functions as a substrate adaptor protein, assembling with CUL5, Rbx2, Elongin B, and Elongin C to form the CRL5 ubiquitin ligase complex. This complex catalyzes K6-linked polyubiquitination of PAICS, which in turn recruits UBAP2, triggering liquid-liquid phase separation and subsequent purinosome assembly. This process enhances the flux of the *de novo* purine synthesis pathway, ultimately promoting cancer cell proliferation (40). The purinosome may thereby exert pro-tumorigenic effects, potentially establishing a self-reinforcing vicious cycle (103, 104).

Additionally, CUL5 interacts with PCMTD2 (Proline-rich coiled-coil maintenance of telomeres 2), a protein involved in maintaining telomere stability (21). In renal cell carcinoma (RCC), CUL5 deficiency disrupts centriole duplication and DNA damage repair mechanisms, leading to genomic instability manifested as chromosomal numerical and structural aberrations. These defects further result in cell cycle dysregulation, driving uncontrolled tumor cell proliferation (12).

## 5 CUL5 maintains proteostasis

### 5.1 CUL5 in the regulation of HSP90 client proteins

Heat shock protein 90 (HSP90), a highly conserved ATP-dependent molecular chaperone, plays critical roles in protein folding, degradation, and trafficking, maintaining proteostasis by directing misfolded or unfolded proteins to the ubiquitin-proteasome system for degradation (105). In cancer, dysregulated HSP90 function is closely associated with tumorigenesis and progression. By stabilizing conformational states of multiple “client proteins,” HSP90 ensures their functional integrity (106). Furthermore, HSP90 can upregulate MHC class I and II molecules (107), enhancing antigen presentation and consequently potentiating cellular immunity (108).

CUL5 cooperates with the HSP90 complex to regulate the stability of oncogenic proteins, such as ERBB2/HER2 (109), which serves as a validated therapeutic target in breast cancer and gastrointestinal malignancies (110). Silencing of CUL5 has been shown to reduce the sensitivity of various cancer cells to HSP90 inhibitors, including 17-AAG and Geldanamycin (42, 111, 112), suggesting a potential role for CUL5 in pathways downstream of HSP90. This notion was substantiated by Cui et al. (113), who demonstrated that in hepatocellular carcinoma (HCC), inhibition of HSP90 significantly promotes the ubiquitination and degradation of the highly expressed RNA polymerase II-associated protein 2 (RPAP2) via the CRL5-FBXW7 E3 ligase complex, thereby suppressing HCC cell proliferation.

### 5.2 CUL5 mediates the specific degradation of aberrant translation products

Okumura et al. (114) discovered that the CUL5-type ubiquitin ligase substrate receptor protein KLHDC1 forms a complex with CUL5. This complex specifically recognizes and degrades the truncated form of selenoprotein S SELENOS(ΔSec) generated by failed decoding of the UGA/Sec termination codon. Importantly, it does not degrade the mature, full-length SELENOS containing intact selenocysteine. The mature SELENOS, through its selenium-dependent oxidoreductase activity, positively regulates intracellular reactive oxygen species (ROS) levels (115). Thus, by clearing inactive truncated proteins, the KLHDC1-CUL5 complex helps maintain cellular proteostasis, indirectly safeguarding the proper function of this redox enzymatic network.

## 6 CUL5 regulates signaling pathways

### 6.1 CUL5 modulation of the JAK pathway

CUL5 has been shown to form ubiquitin E3 ligase complexes with diverse adaptor proteins to mediate degradation of JAK family members (116, 117). Wu et al. (118) demonstrated that NOTCH signaling activation induces transcription of *Asb2* and *Skp2*. These proteins interact to assemble an atypical heterodimeric E3 ubiquitin ligase complex with CUL1 and CUL5, which catalyzes the ubiquitination and degradation of JAK3. A similar regulatory mechanism was identified by Nie et al. for JAK2 (119). In endometrial cancer, low CUL5 expression leads to the accumulation of JAK2 and FAS-L (Fas ligand), driving tumor cell proliferation. This finding underscores the conserved role of CUL5-containing E3 ligase complexes in modulating JAK kinase stability and their impact on oncogenic signaling (13).

### 6.2 CUL5 regulation of the NF-κB/MAPK pathway

CUL5 can bind to its substrate receptor protein SPSB1 to suppress NF-κB activation (120); however, studies exploring this interaction in cancer remain scarce. The TRAF6/NF-κB pathway is well-recognized for its involvement in cancer pathogenesis (121), and CUL5 has been shown to regulate this signaling axis. In LPS-induced sepsis, CUL5 interacts with TRAF6 and promotes its K63-linked polyubiquitination at Lys63, leading to the activation of NF-κB and MAPK pathways, enhanced expression of inflammatory genes, and subsequent production of pro-inflammatory cytokines such as IL-1β, IL-6, and TNF-α (122). In colorectal cancer, LPS binding to TLR4 similarly recruits and activates TRAF6, thereby stimulating the NF-κB pathway, resulting in phosphorylation of p65/RelA and upregulation of VEGF-C expression, which in turn promotes lymphangiogenesis and lymphatic metastasis (123). It is thus plausible that CUL5 may also play a role in the LPS–NF-κB–VEGF-C signaling cascade in tumors, potentially contributing to the regulation of tumor proliferation.

### 6.3 CUL5 regulates autophagy

The mTORC family (mechanistic target of rapamycin complex), comprising mTORC1 and mTORC2 (124), plays pivotal roles in intracellular signaling. In normal mammary epithelial cells (HC11), CUL5 functions as a positive regulator of proliferation. Taurine (Tau) dose-dependently upregulates CUL5 expression through the PI3K pathway, promoting nuclear mTOR mRNA transcription and protein phosphorylation to drive cell growth (125, 126). Conversely, in pancreatic cancer cell lines (PANC1 and BXPC3), the CUL5-SOCS6 complex is reduced, which suppresses the ubiquitination-mediated degradation of Sin1. This leads to increased mTORC2 activity, elevated phosphorylation of AKT, and thereby promotes cell survival and proliferation (44). These findings highlight tissue-specific regulatory mechanisms of CUL5 in mTOR signaling.

The Elongin B/Cullin 5 complex negatively regulates autophagy by ubiquitinating and degrading DEPTOR, an endogenous inhibitor of mTORC1. DEPTOR degradation relieves mTORC1 suppression, thereby inhibiting autophagy. Notably, AMBRA1 binds to Elongin B to inhibit Cullin 5 activity, stabilizing DEPTOR and maintaining mTORC1 inhibition to promote autophagy (127). Studies have confirmed that mTORC1 synergizes with monomeric GTPases during cell proliferation (128), suggesting CUL5 may modulate cell migration through mTORC1-GTPase crosstalk.

In addition to mTORC signaling, CUL5 promotes the ubiquitination and degradation of p62 by forming a complex with ASB6—a protein containing a C-terminal SOCS box motif—thereby enhancing autophagy in hepatocellular carcinoma (129). In bladder cancer, although pan-cancer analyses have revealed reduced CUL5 expression in this malignancy (14), Gao et al. (130) demonstrated that CUL5 deficiency decreases the ubiquitination level of PTBP1. PTBP1 subsequently regulates the alternative splicing of RUBCN pre-mRNA, leading to increased expression of the RUBCN-S isoform, which suppresses autophagy and prevents immune evasion in bladder cancer cells. In xenograft models, CUL5 knockout significantly enhanced the efficacy of anti-PD-1 immunotherapy, thereby inhibiting cell proliferation.

## 7 Regulation of cellular metabolism by CUL5

CUL5 modulates metabolic homeostasis by promoting ubiquitin-mediated degradation of glycolytic, fatty acid metabolic, and amino acid metabolic enzymes (21). CRL5 was shown to cooperate with Alix to maintain mitochondrial proteostasis (131). However, Wang et al. (132) revealed that in cardiomyocytes, RBX2/CRL5 localizes to the mitochondrial outer membrane (MOM), where it ubiquitinates and targets MOM proteins for degradation, thereby promoting mitophagy. Furthermore, RBX2 interacts with PINK1 (PTEN-induced kinase 1) and, via an as-yet-uncharacterized mechanism, impedes its ubiquitination, thereby facilitating the phosphorylation of ubiquitin (pS65-Ub) upon mitochondrial depolarization and enhancing mitophagic flux. In summary, deficiency of RBX2 results in compromised mitochondrial ubiquitination, accumulation of damaged mitochondria, severe mitochondrial dysfunction, culminating in dilated cardiomyopathy and heart failure. This study underscores the pivotal role of CRL5 in maintaining mitochondrial quality and function, suggesting that targeting the mitochondrial RBX2/CUL5 axis may represent a novel therapeutic strategy in oncology.

Studies have confirmed that CUL5 regulates the stability of key proteins involved in cholesterol metabolism through ubiquitination (71). In colorectal cancer (CRC), the CUL5-ASB6 E3 ligase complex has been identified to promote K48-linked ubiquitination of INSIG1, a critical cholesterol regulator, leading to its degradation and subsequent enhancement of cholesterol synthesis. Accumulation of cholesterol supports tumor cell proliferation and survival (133), ultimately driving CRC cell proliferation and metastasis (71).

Increased CRL expression correlates with enhanced ubiquitination of PI3K/AKT signaling substrates and elevated glucose uptake/utilization (18, 35). As the largest member of the CUL family, CUL5 is speculated to participate in this pathway; however, no studies have yet explored the direct regulatory relationship between CUL5/PI3K/AKT and glucose metabolism (134–136), warranting further investigation.

## 8 CUL5 and angiogenesis

CUL5 exerts critical control over tumor angiogenesis by ubiquitinating and modulating key signaling pathways, including TRAF6, HIF, and MAPK. In hypoxic pulmonary hypertension (HPH), elevated CUL5 expression binds TRAF6 (TNF receptor-associated factor 6), activating the NF-κB pathway to stabilize HIF-1α, thereby increasing VEGF expression and driving pulmonary vascular remodeling and HPH progression (39). Thalidomide, an anti-proliferative and anti-angiogenic agent (137), exerts its anti-endothelial effects in normal endothelial cells through CUL5-dependent mechanisms. Reduced nuclear localization of CUL5 correlates with thalidomide resistance, and CUL5 knockdown via siRNA induces endothelial-to-angiogenic phenotype transition (138, 139). Notably, mutating the nuclear localization signal (NLS: 640PKLKRQ646) of CUL5 (e.g., K642G/K644G mutations) significantly diminishes its nuclear translocation, impairing its ability to suppress cell proliferation (9).

CUL5 also suppresses endothelial cell proliferation and angiogenesis by promoting the degradation of Visfatin (NAMPT), thereby inhibiting MAPK/PI3K-AKT signaling (140). Willis et al. (9) demonstrated that CUL5 inhibits pro-angiogenic pathways through ubiquitination and degradation of HIF (hypoxia-inducible factor) and suppression of MAPK phosphorylation. However, the mutant S730A CUL5 reverses this effect by ubiquitin-mediated degradation of maspin—a known anti-angiogenic and tumor suppressor protein—eliminating PKA-dependent phosphorylation sites, enhancing MAPK (Erk1/Erk2) phosphorylation, and promoting Egr-1 nuclear translocation. This activates multiple angiogenesis-related genes, inducing a “pro-angiogenic” phenotype (141).

Thus, in tumor angiogenesis, CUL5 forms a multidimensional regulatory network: it enhances pro-angiogenic signals via the TRAF6/NF-κB/HIF-1α/VEGF axis while simultaneously suppressing the MAPK/PI3K-AKT pathway through Visfatin degradation, balancing HIF stability and MAPK phosphorylation. Beyond angiogenesis, CUL5 indirectly regulates vascular permeability by modulating aquaporins (AQPs). For instance, CUL5 promotes AQP1 degradation via the ubiquitin-proteasome system, reducing cellular water permeability (142). Similarly, CUL5 ubiquitinates AQP2, further influencing vascular dynamics (143, 144), suggesting broader roles in fluid homeostasis during vascular development.

These findings highlight CUL5’s dual role as both a suppressor and promoter of angiogenesis, depending on its interaction partners and post-translational modifications. Targeting CUL5 or its adaptors (e.g., TRAF6, HIF) could offer strategies to normalize tumor vasculature or block pathological angiogenesis in diseases like HPH. Additionally, modulating AQP degradation via CUL5 might provide avenues to control vascular permeability in edema or metastatic settings.

## 9 CUL5 and immune cells

CUL5 modulates immune cell distribution and function by regulating cellular signaling pathways and immune cell activity. Research by Li et al. (14) revealed that in uveal melanoma and head and neck squamous cell carcinoma, CUL5 expression correlates with levels of immune-infiltrating cells within the tumor microenvironment. Deficiency in CUL5 activates the CREB1–CCL2 signaling axis, leading to the accumulation of monocytes and polymorphonuclear myeloid-derived suppressor cells (PMN-MDSCs), accompanied by a reduction in T-cell numbers, thereby fostering an immunosuppressive tumor microenvironment (145). The following sections will elaborate on the detailed regulatory mechanisms through which CUL5 influences different immune cell populations.

### 9.1 CUL5 and T cells

In the majority of malignant tumors, the expression of CUL5 shows a significant negative correlation with the abundance of CD8^+^ T cells, helper T cells, regulatory T cells (Tregs), and Th17 cells, while displaying a positive correlation with central memory T cells (14). The following sections will discuss these relationships by T cell subset.

CUL5 acts as a negative regulator of the core signaling pathway in CD8^+^ T cells. TCR activation significantly upregulates the expression and neddylation of CUL5 (21). CUL5, by interacting with PCMTD2, targets components of the TCR complex (e.g., CD247, also known as CD3ζ) and the IL-2 receptor beta chain (IL2RB) for ubiquitination, leading to their degradation. This process negatively regulates the activation and effector functions of CD8^+^ T cells (21). Regarding the other member of the PCMTD family, PCMTD1, research by Warmack et al. (146) confirmed its interaction with CUL5, although its functional consequences remain unclear.

CUL5 also plays a critical role in CD4+ T cells. Deficiency of CUL5 activates the MAPK/PI3K-AKT signaling pathway, reduces the signaling threshold of IL4R, promotes the differentiation of CD4+ T cells into Th9 and Th2 cells, and inhibits Treg cell differentiation. This is significant for the development of allergic conditions such as asthma (147). Similarly, a shift from Th1 to Th2 cells is often observed in malignant tumors (148). In Tregs, increased expression of GRAIL, a RING E3 ubiquitin protein ligase, mediates ubiquitination of CUL5 at a specific site (K724) involved in its neddylation. This modification blocks the activation of CUL5 CRLs, reduces the ubiquitination-mediated degradation of pJAK1 and Deptor, and suppresses the negative regulation of IL-2R signaling, thereby enhancing Treg function (149, 150).

In Tregs, increased expression of the RING E3 ubiquitin protein ligase GRAIL leads to the ubiquitination of a specific lysine residue (K724) on Cul5. This residue is critical for Cul5 neddylation, and its modification by GRAIL blocks the activation of Cul5-containing CRLs. This inhibition reduces the ubiquitin-mediated degradation of targets like phosphorylated JAK1 (pJAK1) and Deptor, thereby attenuating the negative regulation of the IL-2 receptor signaling pathway. The net effect is an enhancement of Treg function (151, 152). In the future, more precise tumor cell therapies could be achieved by utilizing nanocarriers, adeno-associated virus (AAV), or lentiviral vectors to deliver functional CUL5 genes to T cells within the tumor microenvironment, or by integrating these approaches into CAR-T cell delivery platforms (153, 154)

### 9.2 CUL5 and other immune cells

Respiratory viral infections upregulate CUL5 expression, which correlates with increased neutrophil infiltration. Conversely, CUL5 deficiency ameliorates neutrophilic pulmonary inflammation by enhancing IFN-β production (72). Mechanistically, CUL5 interacts with O-GlcNAc transferase (OGT) to induce K48-linked polyubiquitination of OGT. This modification blocks OGT-mediated O-GlcNAcylation of mitochondrial antiviral signaling protein (MAVS) and RIG-I, thereby suppressing RIG-I signaling activation and negatively regulating IFN-β expression. It is plausible that in tumors, CUL5 may modulate immune cell phenotypes and functions by ubiquitin-mediated degradation of pro-inflammatory factors or their signaling molecules. For instance, Chen et al. (145) demonstrated that Cul5 deficiency elevates levels of chemokines such as CCL2, CCL5, CXCL5, and CXCL10, which are critical for monocyte recruitment (149, 150, 155–158).

CUL5 deficiency promotes megakaryopoiesis largely independent of the thrombopoietin/MPL pathway, instead relying on signaling through the β−common (βc) and/or β−IL-3 (βIL-3) receptors (81, 159). Using a hematopoietic-specific conditional knockout mouse model (Cul5^+^/Vav-Cre), Tomishima et al. (160) demonstrated that loss of CUL5 leads to hyperactivation of hematopoietic stem cells (HSCs) and a bias toward myeloid and megakaryocyte differentiation. This phenotype is driven by the failure of CUL5 to negatively regulate IL-3-induced STAT5 signaling via the CRL5-LRRC41 complex, which is essential for maintaining HSC quiescence and lineage balance. Collectively, these findings establish Cul5 as a critical negative regulator of IL-3 signaling and a key guardian of hematopoietic homeostasis.

## 10 Dual regulatory roles of CUL5 in cancer

In various cancers [e.g., breast cancer (11), renal cancer (12), and endometrial carcinoma (13)], CUL5 expression is downregulated (13). For instance, in breast cancer, Talamantez-Lyburn et al. (19) confirmed low CUL5 expression in ErbB2+ tumors, which may be associated with frequent deletions at chromosomal region 11q22-23, the genomic locus of CUL5 (7). Overexpression of CUL5 in breast cancer suppresses MAPK phosphorylation and estrogen-dependent cell growth (139). Furthermore, the CRL5 complex can prevent Src-induced transformation of mammary epithelial cells by promoting the ubiquitination and degradation of p130Cas (also known as Breast Cancer Anti-estrogen Resistance protein 1, BCAR1) (161). These findings suggest that strategies aimed at enhancing CRL5 activity could serve as a component of combination therapy, particularly for ErbB2+ or endocrine-resistant breast cancers.

Conversely, CUL5 expression is elevated in a subset of cancers, such as colorectal carcinoma (15) and hepatitis B virus-induced hepatocellular carcinoma (16). For example, in colorectal cancer, CUL5 interacts with Endoplasmic Reticulum Protein 29 (ERp29), a protein involved in endoplasmic reticulum stress (ERS), and their cooperation promotes cancer cell invasion, migration, and epithelial-mesenchymal transition (15).

Additionally, CUL5 expression holds prognostic value. Low CUL5 levels are associated with poorer prognosis in cholangiocarcinoma, renal clear cell carcinoma, rectal adenocarcinoma, and adrenocortical carcinoma. In contrast, high CUL5 expression correlates with adverse outcomes in adrenocortical carcinoma and colorectal cancer (14, 15).

In summary, the expression and function of CUL5 in cancer are highly context-dependent. This dual, seemingly paradoxical role of CUL5 across different tumor types warrants further investigation. The expression levels, regulatory mechanisms, downstream effects, and therapeutic strategies of CUL5 in various cancers are summarized in Table 1.

**TABLE 1:** Summary of CUL5 expression levels, regulatory mechanisms, downstream effects, and therapeutic strategies in various cancers.

Cancer type	Expression level	Mechanisms regulating CUL5	Downstream effects resulting from CUL5 expression levels	Therapeutic strategy	Evidence types for CUL5 regulatory mechanisms	References
Renal cell carcinoma	Low	Unknown	Centriole overduplication; increased multipolar and pseudo-bipolar mitoses	None	Observational correlation studies	(12)
Breast cancer	Low	Unknown	Resistance to HSP90 inhibitor (17-AAG)	Glutathione (GSH)-coated gold nanoparticles (AuNPs) co-loaded with CUL5 DNA and 17-AAG	Observational correlation studies	(19)
Breast cancer	Low	Unknown	Reduced ubiquitination and degradation of CREB1; increased secretion of CCL2 and other cytokines; formation of immunosuppressive tumor microenvironment	CREB1 inhibitor 666-15	Observational correlation studies	(145)
		Unknown	Rab40b/Cul5 complex regulates Rap2 localization and activity via ubiquitination, promoting breast cancer cell migration	None	Not applicable	(60)
Hepatocellular carcinoma	HBV-related high	Hepatitis B virus X protein downregulates miR-145, leading to CUL5 upregulation	Promotion of G0/G1→G2/M phase transition; inhibition of apoptosis	None	Functional/mechanistic studies	(16)
	Non-viral low	Low miR-7 levels induce CUL5 downregulation	Promotion of G1/S phase transition in tumor cells	None	Functional/mechanistic studies	(165)
Colorectal cancer	High	circINSIG1 recruits CUL5-ASB6	Promotes cholesterol synthesis and CRC progression	None	Not applicable	(71, 169)
Colorectal cancer	High	Endoplasmic reticulum stress (ERS) induces CUL5 expression	ERp29 synergizes with CUL5 to restore cell invasion and migration, promoting epithelial-mesenchymal transition (EMT)	siRNA-mediated CUL5 knockdown	Functional/mechanistic studies	(15)
Non-small cell lung cancer	Unknown	UBE2F promotes CUL5 neddylation	Ubiquitination of NOXA; enhanced lung cancer cell survival	HA-9104 selectively inhibits CUL5 neddylation	Not applicable	(91, 92)
Small cell lung cancer	Low	Unknown	Integrin β1 accumulation activates FAK/SRC signaling, promoting tumor metastasis	Dasatinib (SRC inhibitor)	Not applicable	(46)
Endometrial cancer	Low	miR-182 suppresses CUL5	Increased JAK2/FAS-L expression	None	Functional/mechanistic studies	(13)
Gastric cancer	Low	miR-19a inhibits CUL5	activates PI3K/AKT/ERK pathways, and promotes proliferation/metastasis	None	Observational correlation studies	(163)
Prostate cancer	Low	DCUN1D1 overexpression enhances neddylation of CUL1/3/4A/5	CUL5 neddylation activates Wnt/β-catenin signaling	None	Not applicable	(84)
		miR-19a represses CUL5.	Reduced CUL5 expression promotes EMT and metastasis	None	Functional/mechanistic studies	(164)
Pancreatic cancer	Unknown	UBE2F-mediated neddylation activates CRL5	Ubiquitination and degradation of DIRAS2; activation of MAPK-c-Myc signaling pathway; enhanced tumor cell proliferation	MLN4924 (NAE inhibitor) inhibits CUL5 neddylation	Not applicable	(87)
		Reduced CUL5-SOCS6 complex formation	Accumulation of Sin1; inhibition of mTORC2-AKT signaling pathway	None	Not applicable	(44)
Bladder cancer	Low	Unknown	Decreased ubiquitination of PTBP1, leading to elevated levels of the short RUBCN isoform (RUBCN-S), which suppresses autophagy and prevents immune evasion of cancer cells.	None	Not applicable	(130)

### 10.1 Post-transcriptional regulatory mechanisms of CUL5 in tumors: from miRNAs to circRNAs

MicroRNAs (miRNAs), a class of small non-coding RNAs, negatively regulate CUL5 expression by binding to the 3’ untranslated region (3’UTR) of CUL5 mRNA, forming RNA-induced silencing complexes (RISCs) that inhibit translation or induce mRNA degradation (162). In endometrial cancer, CUL5 is significantly downregulated, with miR-182 identified as a key regulator. MiR-182 suppresses CUL5 translation or promotes its degradation, leading to abnormal accumulation of downstream JAK2 and Fas-L, which drives tumor cell proliferation (13).

In gastric cancer, Zhu et al. (163) demonstrated that miR-19a inhibits CUL5 expression, thereby relieving CUL5-mediated suppression of downstream targets and activating the PI3K/AKT pathway. This results in elevated phosphorylated AKT (p-AKT) and p-ERK levels, promoting gastric cancer cell proliferation, migration, and invasion. In prostate cancer, upregulated miRNA-19a has been shown to repress CUL5, thereby promoting epithelial-mesenchymal transition (EMT) and metastasis of prostate cancer cells (164).

Gao et al. (16) demonstrated that the hepatitis B virus X protein (HBx) downregulates miR-145, leading to upregulation of CUL5, which promotes cell cycle progression (from G0/G1 to G2/M) and inhibits apoptosis. Similarly, in QGY-7703 and HepG2 hepatocellular carcinoma cell lines, downregulation of miR-7 attenuates CUL5 expression, thereby promoting HCC cell proliferation and G1/S phase transition (165). These findings indicate that the regulatory effects of distinct miRNAs on CUL5 vary across different tumor types and may be either positive or negative, underscoring the necessity of case-by-case investigation for specific miRNAs.

Satoko Nakashima et al. (166) reported that downregulation of circ_0024169 in angiosarcoma, and suggested that the circ_0024169/CUL5 ratio may serve as a diagnostic biomarker for vascular tumors. We speculate that the sponge effect of circ_0024169 (167)—whereby circRNA competitively binds miRNAs via complementary sequences, thereby inhibiting their regulatory activity on target genes—may enhance the activity of certain miRNAs, leading to suppressed CUL5 expression. In colorectal cancer (CRC) tissues, a novel hypoxia-responsive circular RNA (circRNA), circINSIG1, has been identified. Its encoded protein, circINSIG1-121, recruits the CUL5-ASB6 E3 ligase complex to promote K48-linked ubiquitination of INSIG1—a key regulator of cholesterol metabolism—at lysine residues 156 and 158, thereby inducing cholesterol synthesis and subsequently facilitating CRC cell proliferation and metastasis (71). Given that circRNA levels are frequently downregulated in various tumor tissues, the circRNA/CUL5 ratio may serve as a diagnostic biomarker for multiple cancer types (168). Furthermore, distinct CUL5-targeting miRNAs exhibit differential expression across tumors and are inversely correlated with CUL5 expression levels. Therefore, assessing specific miRNA expression profiles or the circRNA/CUL5 ratio may offer novel strategies and biomarkers for early diagnosis, molecular subtyping, and personalized targeted therapy in diverse cancers.

## 11 Challenges and future directions in targeting CUL5 for tumor cell therapy

### 11.1 Limitations and advances in existing pharmacological intervention strategies

Currently, few drugs directly target CUL5. However, its function can be indirectly modulated by interfering with its neddylation. For example, gossypol non-specifically blocks the neddylation of both CUL5 and CUL1 (17, 18), while HA-9104 specifically inhibits UBE2F, thereby selectively suppressing CUL5 neddylation (92). Lubbers et al. (170) found that treating breast cancer cells with resveratrol, a natural dietary component, significantly increased CUL5 expression and inhibited the growth of T47D breast cancer cells. Jia et al. (11) demonstrated that interferon-gamma (IFN-γ) could upregulate CUL5 expression and disrupt the binding of Cdc37/Hsp90 to HER2, consequently inducing tumor cell senescence and promoting HER2 degradation. Kabir et al. (171) discovered that CUL5 knockout significantly sensitized lung cancer cells to CDK9 and MCL1 inhibitors, suggesting that targeting CUL5 could overcome drug resistance in these cells.

Furthermore, combining strategies that target CUL5 with other treatments, such as neddylation inhibitors (88), chemotherapy (137), radiotherapy (92), or other targeted therapies (171), may synergistically enhance anticancer efficacy and overcome resistance mechanisms.

### 11.2 Application of nanotechnology in CUL5-targeted therapy

Given that CUL5 is widely involved in normal cellular physiological functions and its neddylation pathway partially overlaps with other Cullin family members (e.g., CUL1), existing targeting strategies often suffer from suboptimal specificity and are prone to off-target effects (18, 172). Therefore, there is an urgent need to develop inhibitors with enhanced targeting precision. Nanotechnology provides a promising platform for achieving more accurate targeting of CUL5 (173). Currently, direct experimental studies applying nanotechnology to specifically modulate CUL5 are relatively limited (20) and remain at an early exploratory stage. This section systematically reviews and draws analogies to nanotechnology strategies that have demonstrated progress for other targets or disease models, based on shared mechanistic principles. It aims to explore the potential feasibility and future directions of adapting these advanced technological platforms for CUL5-targeted therapy.

#### 11.2.1 Gold nanoparticles

To date, the only reported application of CUL5-targeted nanomedicine involves gold nanoparticles (AuNPs) loaded with CUL5-encoding DNA. Talamantez-Lyburn et al. (19) demonstrated that delivery of these AuNPs into CUL5-deficient breast cancer cells significantly increased CUL5 expression, restored sensitivity to the HSP90 inhibitor 17-AAG, and enhanced apoptosis. Although unmodified inorganic AuNPs offer the advantage of high structural stability (174), they exhibit limitations in targeting specificity and intracellular trafficking efficiency (175). Surface functionalization of AuNPs with single or multiple, identical or distinct, tumor-specific antigens or receptor ligands—such as peptide-based targeting moieties, monosaccharides, or biocompatible coatings [e.g., polyethylene glycol (PEG)]—has been extensively validated in other disease models to substantially overcome these limitations (176–179).

#### 11.2.2 Lipid nanoparticles (LNPs)—a platform with potential for CUL5 targeting

Lipid nanoparticles (LNPs) are an established clinical platform for nucleic acid delivery, capable of efficiently encapsulating and protecting siRNA or mRNA and enabling intracellular release through modulation of lipid composition (180, 181). In principle, LNPs could be employed to suppress CUL5 expression via CUL5-targeting siRNA or to modulate immune cells through mRNA encoding chimeric antigen receptors (CARs) or CUL5-regulatory molecules (182, 183).

Furthermore, targeting precision may be enhanced through various strategies, including high-throughput screening (HTS) to identify LNP formulations with specific organ tropism (184), cell membrane-coated nanoparticles (CMNPs) (185), and the design of triggerable release systems (186, 187), among others.

Of particular relevance to cancer cell therapy, LNPs surface-functionalized with antibody fragments targeting CD5 (a T-cell surface marker) and encapsulating CAR-encoding mRNA have enabled direct in vivo CAR gene modification of T cells, circumventing the need for complex *ex vivo* manufacturing (181). Billingsley et al. (188) further developed an antibody-conjugated LNP (Ab-LNP) platform that achieved robust *in vivo* T-cell transfection and generated functional CAR-T cells capable of eliminating B-cell lymphoma in murine models. These validated proof-of-concept studies suggest that Ab-LNP-mediated delivery of CUL5-regulatory mRNA to enhance T-cell activation and proliferation may be feasible, although this remains to be experimentally demonstrated for CUL5 specifically.

#### 11.2.3 Polymeric nanoparticles (PNPs)—a platform with established precedent, lacking CUL5-Specific evidence

Conventional polymeric nanoparticles (PNPs) are composed of a polymer backbone conjugated with therapeutic agents (182). Incorporating chemical linkages between the ligand and the backbone that are cleavable within the tumor microenvironment enables site-specific drug release or ligand exposure at the disease site (183). PNPs have been extensively validated in preclinical cancer models for non-CUL5 payloads (189). With particle sizes of approximately 100–200 nm, they leverage the enhanced permeability and retention (EPR) effect for passive targeting and accumulation in tumor tissues (190). High-throughput fabrication methods, including microfluidics and template assembly (191), further enable the generation of complex nanostructures with uniform morphology. However, no studies to date have reported PNP-based delivery of CUL5-targeted agents. Proof-of-concept validation in appropriate CUL5-dependent tumor models is urgently warranted.

### 11.3 The regulatory role of CUL5 in cellular immunotherapy

Adoptive cell therapy (ACT) is a cornerstone of cancer cell therapy, involving the infusion of *ex vivo* activated, expanded, or genetically engineered immune cells back into patients to enhance anti-tumor immunity (192). CUL5 has been found to influence the efficacy of these therapies.

#### 11 3.1 CAR-T/TCR-T/CAR-NK therapies

**FIGURE 2:**
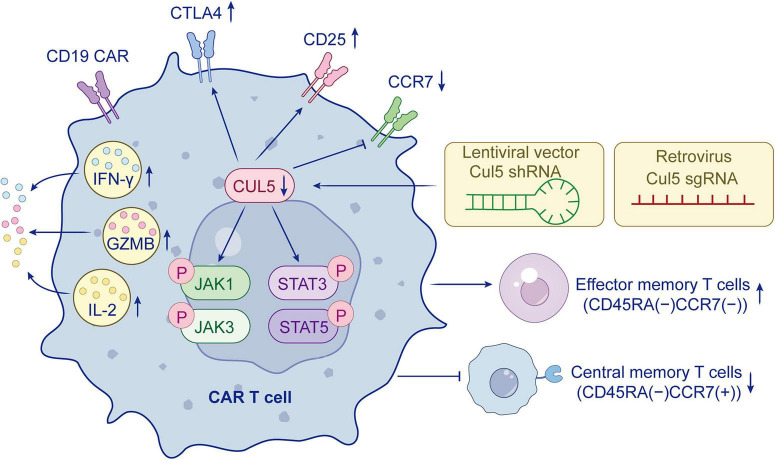
Regulation and downstream effects of CUL5 in CAR-T cells.

CAR-T, TCR-T, and CAR-NK therapies confer specific antigen recognition capabilities to immune cells through viral vectors or gene-editing technologies for precise targeting. CUL5 deficiency has been shown to significantly enhance the proliferative capacity of CAR T cells. CD19 CAR T cells with CUL5 knockout exhibit sustained phosphorylation of STAT3 and STAT5, as well as delayed phosphorylation and degradation of JAK1 and JAK3. These molecular changes enhance the expansion potential and effector function of human CD19 CAR T cells, leading to increased secretion of cytokines such as interferon-γ (IFN-γ) and interleukin-2 (IL-2), thereby augmenting their anti-tumor potency (20). Concurrently, CUL5-deficient CAR T cells exhibit an increased proportion of effector memory T cell (TEM) phenotype and a reduced central memory T cell (TCM) phenotype, which may contribute to improved anti-tumor efficacy. Researchers also observed that CUL5 knockout leads to a significant increase in CTLA4 expression and a decrease in PD-1 expression (21). CTLA4 is a key immune checkpoint molecule on the T cell surface. Its high expression and subsequent binding to CD80/CD86 on antigen-presenting cells can inhibit excessive T-cell activation and dampen anti-tumor efficacy (193). Its upregulation may therefore serve as an intrinsic feedback mechanism to prevent exhaustion or toxicity caused by overactivation. By simultaneously knocking out both CTLA4 and CUL5, they found that the anti-tumor capacity of T cells could be further enhanced. This suggests that knocking out CUL5 in human CD8^+^ CAR T cells similarly enhances CAR signaling activity, IFN-γ secretion, and tumor cell clearance capability (22). These findings provide a novel strategy for CAR-T cell-based cancer therapy (21). This suggests that knocking out CUL5 in human CD8^+^ CAR T cells similarly enhances CAR signaling activity, IFN-γ secretion, and tumor cell clearance capability. These observations provide a novel strategy for CAR-T cell-based cancer therapy (Figure 2).

Despite the immense potential of CAR-T cells, they carry risks such as on-target/off-tumor toxicity. Researchers are exploring methods to remotely control CAR-T cell activation to mitigate these risks, including heat shock or ultrasound-induced activation (194). For instance, Tang et al. (195) constructed HS-CAR-T cells incorporating a 7HE heat shock promoter, which restricts CAR expression and activation to heated areas/time periods, thereby reducing off-target toxicity and potentially preventing T cell exhaustion. As CUL5 is an intracellular E3 ubiquitin ligase component rather than a membrane protein, a similar strategy could be envisioned by linking the 7HE promoter to a CUL5-specific TCR, creating a heat-inducible TCR-T system. Furthermore, studies by Verma et al. (196) found that knocking out CUL5 in TCR-T cells increases their proliferation rate and enhances anti-cancer responses. Future approaches may combine these strategies, for example, by knocking out the endogenous CUL5 gene in CUL5-targeting TCR-T cells. This could simultaneously enhance T cell function and potentially prevent fratricide among the engineered T cells.

Engineered NK cells expressing chimeric antigen receptors (CARs) have demonstrated potent anti-tumor efficacy in both preclinical and clinical studies (197). In various cancers, the expression of CUL5 exhibits a negative correlation with the level of NK cell immune infiltration (14). Utilizing CRISPR/Cas9 technology to knockout genes such as *CUL5* and *UBE2F*, Nikolic et al. (22) aimed to enhance the IL-15R signaling pathway. Following multiple rounds of *in vitro* and *in vivo* challenges, the gene-edited CAR-NK cells maintained significantly higher cytotoxicity against BCMA-positive tumor cells compared to control cells. Future research could further explore the combination of CUL5-knockout CAR-NK cells with other immunotherapeutics, such as PD-1/PD-L1 inhibitors, to potentially amplify the anti-tumor response.

Furthermore, the approaches mentioned above can be extended. For instance, peptides or proteins mimicking the SOCS box could be developed. These mimics would exploit the molecular assembly mechanism between SOCS-box proteins and the CUL5-Elongin BC complex (32), acting as competitive inhibitors to disrupt CUL5 function. Alternatively, targeting other critical components within the CRL5 architecture, such as RBX2, might achieve equivalent and potentially more precise regulation.

#### 11.3.2 Macrophage therapy and dendritic cell (DC) therapy

A major obstacle for T cell-based therapies is their infiltration into solid tumors. Given their inherent and sustained migratory capacity toward inflammatory and tumor sites, mononuclear phagocytes, particularly macrophages, offer a potential strategy to overcome this limitation (198). In asthma patients, high expression of Cullin5 in alveolar macrophages suppresses IFN-β production and impairs antiviral immunity (72). Knocking out the negative regulator CUL5 in macrophages may therefore enhance their antiviral or anti-tumor functions. Furthermore, CUL5-deficient macrophages exhibit a reduced activation threshold in response to IL-4, characterized by increased arginase 1 expression and a marked shift toward an anti-inflammatory phenotype, with upregulation of anti-inflammatory proteins such as Serpinb6a and Aldh5a1 (199). Building on these findings, integrating CUL5 modulation strategies with existing CAR-M (chimeric antigen receptor macrophage) therapies holds promise for developing next-generation engineered macrophages with enhanced tumor-killing capacity and microenvironment-remodeling abilities.

Dendritic cell vaccines involve loading DCs with tumor antigens *ex vivo* before reinfusing them into patients to induce tumor-specific T cell responses (200). Li et al. (14) found a negative correlation between CUL5 expression levels and the infiltration of plasmacytoid dendritic cells (pDCs) in tumors. Since DC maturation and function are regulated by pathways such as JAK and MAPK (201), and CUL5 is known to modulate these key signaling cascades (116, 117, 125, 126), theoretically, modulating CUL5 could alter the immunostimulatory capacity of DCs within the tumor microenvironment.

#### 11.3.3 potential risks and challenges of modulating CUL5 in cellular immunotherapies

CUL5 is widely expressed in the human body (139) and participates in numerous physiological functions, including the regulation of cell proliferation and the cell cycle (50), DNA damage response and repair (102, 202), maintenance of proteostasis, and signal transduction (119). Although preclinical studies highlight the efficacy benefits of modulating CUL5 in enhancing immune cell function, translating these strategies to clinical application requires careful consideration of the following potential—and likely more—risks and limitations. (1). Immune homeostasis dysregulation and autoimmunity risk: as CUL5 is a key negative feedback regulator of both T-cell receptor (TCR) and cytokine signaling pathways (21), its excessive inhibition may lead to hyperactivation of T-cell signaling, breaking immune tolerance and increasing the risk of attacking normal tissues, thereby potentially triggering autoimmune diseases or severe inflammatory responses. (2). Impact on T-cell differentiation: although CUL5 deletion promotes an effector memory phenotype and enhances short-term function (21), its long-term effects on T cells remain unclear. (3). Genomic instability and malignant transformation risk: CUL5 plays a role in maintaining genomic stability and regulating DNA damage repair (12). Genetic knockout of CUL5 in long-lived therapeutic cells could, in theory, increase the risk of genomic instability or even malignant transformation over time—a risk that necessitates thorough long-term investigation. (4). Lack of controllability in modulation: a major technical bottleneck *in vivo* cell therapies is achieving controllable, reversible, and spatiotemporally specific regulation of CUL5 expression or activity (203). Sustained inhibition of CUL5 after tumor clearance could be detrimental.

## 12 Conclusion

CUL5, as the core scaffold protein of the Cullin-RING ligase 5 (CRL5) complex, plays a crucial role in the initiation and progression of malignant tumors by regulating the stability of diverse substrate proteins through the ubiquitin-proteasome system. The activity of CUL5 is highly dependent on NEDD8-mediated neddylation. Its aberrant activation or inhibition can influence tumor cell proliferation, migration, apoptosis, and metabolic reprogramming by modulating key molecules such as NOXA, mTORC, and TRAF6/NF-κB. Furthermore, CUL5 participates in shaping the tumor microenvironment and regulating anti-tumor immunity. Despite being a pivotal regulatory node in tumor cell therapy response, non-selective inhibition of CUL5 may trigger undesirable off-target effects. The dual, context-dependent role of CUL5 in various cancers highlights the necessity of considering specific tumor backgrounds for its clinical application. Existing strategies, such as using gossypol or resveratrol, lack specificity. Emerging nanotechnologies offer promising platforms for achieving precise targeting. Directly modulating CUL5 expression in cellular immunotherapies has demonstrated significant potential. Future directions may involve integrating gene-editing and nanotechnology approaches to precisely regulate CUL5 within immune cells, aiming to overcome critical bottlenecks in solid tumor therapy.

## References

[1] Kim YJ Lee Y Shin H Hwang S Park J Song EJ. Ubiquitin-proteasome system as a target for anticancer treatment-an update. *Arch Pharm Res* (2023) 46:573–97. 10.1007/s12272-023-01455-0 37541992

[2] Sampson C Wang Q Otkur W Zhao H Lu Y Liu X et al. The roles of E3 ubiquitin ligases in cancer progression and targeted therapy. *Clin Transl Med* (2023) 13:e1204. 10.1002/ctm2.1204 36881608 PMC9991012

[3] Soucy TA Smith PG Milhollen MA Berger AJ Gavin JM Adhikari S et al. An inhibitor of NEDD8-activating enzyme as a new approach to treat cancer. *Nature* (2009) 458:732–6. 10.1038/nature07884 19360080

[4] Jang SM Redon CE Aladjem MI. Chromatin-bound cullin-ring ligases: regulatory roles in DNA replication and potential targeting for cancer therapy. *Front Mol Biosci.* (2018) 5:19. 10.3389/fmolb.2018.00019 29594129 PMC5859106

[5] Deshaies RJ Joazeiro CA. RING domain E3 ubiquitin ligases. *Annu Rev Biochem.* (2009) 78:399–434. 10.1146/annurev.biochem.78.101807.093809 19489725

[6] Burnatowska-Hledin MA Spielman WS Smith WL Shi P Meyer JM Dewitt DL. Expression cloning of an AVP-activated, calcium-mobilizing receptor from rabbit kidney medulla. *Am J Physiol.* (1995) 268:F1198–210. 10.1152/ajprenal.1995.268.6.f1198 7611460

[7] Byrd PJ Stankovic T McConville CM Smith AD Cooper PR Taylor AM. Identification and analysis of expression of human VACM-1, a cullin gene family member located on chromosome 11q22-23. *Genome Res.* (1997) 7:71–5. 10.1101/gr.7.1.71 9037604

[8] Ceremuga TE Yao XL Xia Y Mukherjee D McCabe JT. Osmotic stress increases cullin-5 (cul-5) mRNA in the rat cerebral cortex, hypothalamus and kidney. *Neurosci Res.* (2003) 45:305–11. 10.1016/s0168-0102(02)00228-6 12631466

[9] Willis AN Dean SE Habbouche JA Kempers BT Ludwig ML Sayfie AD et al. Nuclear localization signal sequence is required for VACM-1/CUL5-dependent regulation of cellular growth. *Cell Tissue Res.* (2017) 368:105–14. 10.1007/s00441-016-2522-7 27834018

[10] Lamsoul I Uttenweiler-Joseph S Moog-Lutz C Lutz PG. Cullin 5-RING E3 ubiquitin ligases, new therapeutic targets? *Biochimie.* (2016) 122:339–47. 10.1016/j.biochi.2015.08.003 26253693

[11] Jia Y Kodumudi KN Ramamoorthi G Basu A Snyder C Wiener D et al. Th1 cytokine interferon gamma improves response in HER2 breast cancer by modulating the ubiquitin proteasomal pathway. *Mol Ther.* (2021) 29:1541–56. 10.1016/j.ymthe.2020.12.037 33412308 PMC8058490

[12] Tapia-Laliena M Korzeniewski N Peña-Llopis S Scholl C Fröhling S Hohenfellner M et al. Cullin 5 is a novel candidate tumor suppressor in renal cell carcinoma involved in the maintenance of genome stability. *Oncogenesis.* (2019) 8:4. 10.1038/s41389-018-0110-2 30631037 PMC6328621

[13] Devor EJ Schickling BM Reyes HD Warrier A Lindsay B Goodheart MJ et al. Cullin-5, a ubiquitin ligase scaffold protein, is significantly underexpressed in endometrial adenocarcinomas and is a target of miR-182. *Oncol Rep.* (2016) 35:2461–5. 10.3892/or.2016.4605 26847831 PMC4774736

[14] Li Z Hu N Dai L Hou X Hu W Liang W et al. Cullin-5 (CUL5) as a potential prognostic marker in a pan-cancer analysis of human tumors. *Bioengineered.* (2021) 12:5348–60. 10.1080/21655979.2021.1940042 34415831 PMC8806887

[15] Guo L Ma L Liu C Lei Y Tang N Huang Y et al. ERp29 counteracts the suppression of malignancy mediated by endoplasmic reticulum stress and promotes the metastasis of colorectal cancer. *Oncol Rep.* (2019) 41:1603–15. 10.3892/or.2018.6943 30569094 PMC6365697

[16] Gao F Sun X Wang L Tang S Yan C. Downregulation of microRNA-145 caused by hepatitis B virus x protein promotes expression of CUL5 and contributes to pathogenesis of hepatitis B virus-associated hepatocellular carcinoma. *Cell Physiol Biochem.* (2015) 37:1547–59. 10.1159/000438522 26512974

[17] Yu Q Hu Z Shen Y Jiang Y Pan P Hou T et al. Gossypol inhibits cullin neddylation by targeting SAG-CUL5 and RBX1-CUL1 complexes. *Neoplasia.* (2020) 22:179–91. 10.1016/j.neo.2020.02.003 32145688 PMC7076571

[18] Yu Q Sun Y. Targeting protein neddylation to inactivate cullin-RING ligases by gossypol: a lucky hit or a new start? *Drug Des Devel Ther.* (2021) 15:1–8. 10.2147/dddt.s286373 33442232 PMC7797302

[19] Talamantez-Lyburn S Brown P Hondrogiannis N Ratliff J Wicks SL Nana N et al. Gold nanoparticles loaded with cullin-5 DNA increase sensitivity to 17-AAG in cullin-5 deficient breast cancer cells. *Int J Pharm.* (2019) 564:281–92. 10.1016/j.ijpharm.2019.04.022 30999048 PMC6584956

[20] Adachi Y Terakura S Osaki M Okuno Y Sato Y Sagou K et al. Cullin-5 deficiency promotes chimeric antigen receptor T cell effector functions potentially via the modulation of JAK/STAT signaling pathway. *Nat Commun.* (2024) 15:10376. 10.1038/s41467-024-54794-x 39658572 PMC11631977

[21] Liao X Li W Zhou H Rajendran BK Li A Ren J et al. The CUL5 E3 ligase complex negatively regulates central signaling pathways in CD8(+) T cells. *Nat Commun.* (2024) 15:603. 10.1038/s41467-024-44885-0 38242867 PMC10798966

[22] Nikolic I Cursons J Shields B Chappaz S Sudholz H Meng X et al. Enhancing anti-tumor immunity of natural killer cells through targeting IL-15R signaling. *Cancer Cell.* (2025) 43:2034.e–50.e. 10.1016/j.ccell.2025.05.011 40513576

[23] Liu B Zhou H Tan L Siu KTH Guan XY. Exploring treatment options in cancer: tumor treatment strategies. *Signal Transduct Target Ther.* (2024) 9:175. 10.1038/s41392-024-01856-7 39013849 PMC11252281

[24] Bullock AN Debreczeni JE Edwards AM Sundström M Knapp S. Crystal structure of the SOCS2-elongin C-elongin B complex defines a prototypical SOCS box ubiquitin ligase. *Proc Natl Acad Sci U S A.* (2006) 103:7637–42. 10.1073/pnas.0601638103 16675548 PMC1472497

[25] Lumpkin RJ Baker RW Leschziner AE Komives EA. Structure and dynamics of the ASB9 CUL-RING E3 Ligase. *Nat Commun.* (2020) 11:2866. 10.1038/s41467-020-16499-9 32513959 PMC7280518

[26] Yu X Yu Y Liu B Luo K Kong W Mao P et al. Induction of APOBEC3G ubiquitination and degradation by an HIV-1 Vif-Cul5-SCF complex. *Science.* (2003) 302:1056–60. 10.1126/science.1089591 14564014

[27] Mehle A Thomas ER Rajendran KS Gabuzda D. A zinc-binding region in Vif binds Cul5 and determines cullin selection. *J Biol Chem.* (2006) 281:17259–65. 10.1074/jbc.m602413200 16636053

[28] Zheng N Schulman BA Song L Miller JJ Jeffrey PD Wang P et al. Structure of the Cul1-Rbx1-Skp1-F boxSkp2 SCF ubiquitin ligase complex. *Nature.* (2002) 416:703–9. 10.2210/pdb1ldd/pdb 11961546

[29] Zhuang M Calabrese MF Liu J Waddell MB Nourse A Hammel M et al. Structures of SPOP-substrate complexes: insights into molecular architectures of BTB-Cul3 ubiquitin ligases. *Mol Cell.* (2009) 36:39–50. 10.1016/j.molcel.2009.09.022 19818708 PMC2847577

[30] Jang SM Redon CE Thakur BL Bahta MK Aladjem MI. Regulation of cell cycle drivers by Cullin-RING ubiquitin ligases. *Exp Mol Med.* (2020) 52:1637–51. 10.1038/s12276-020-00508-4 33005013 PMC8080560

[31] Skaar JR Pagan JK Pagano M. Mechanisms and function of substrate recruitment by F-box proteins. *Nat Rev Mol Cell Biol.* (2013) 14:369–81. 10.1038/nrm3582 23657496 PMC3827686

[32] Kanno H Matsumoto S Yoshizumi T Nakahara K Kubo A Murata H et al. Role of SOCS and VHL proteins in neuronal differentiation and development. *Int J Mol Sci.* (2023) 24:3880. 10.3390/ijms24043880 36835292 PMC9960776

[33] Kandala S Kim IM Su H. Neddylation and deneddylation in cardiac biology. *Am J Cardiovasc Dis.* (2014) 4:140–58. 10.1038/ncb1301 25628956 PMC4299693

[34] Kamura T Maenaka K Kotoshiba S Matsumoto M Kohda D Conaway RC et al. VHL-box and SOCS-box domains determine binding specificity for Cul2-Rbx1 and Cul5-Rbx2 modules of ubiquitin ligases. *Genes Dev.* (2004) 18:3055–65. 10.1101/gad.1252404 15601820 PMC535916

[35] Wang W Shi B Cong R Hao M Peng Y Yang H et al. RING-finger E3 ligases regulatory network in PI3K/AKT-mediated glucose metabolism. *Cell Death Discov.* (2022) 8:372. 10.1038/s41420-022-01162-7 36002460 PMC9402544

[36] Chen SH Jang GM Hüttenhain R Gordon DE Du D Newton BW et al. CRL4(AMBRA1) targets Elongin C for ubiquitination and degradation to modulate CRL5 signaling. *EMBO J.* (2018) 37:e97508. 10.15252/embj.201797508 30166453 PMC6138441

[37] Zhou W Xu J Tan M Li H Li H Wei W et al. UBE2M is a stress-inducible dual E2 for neddylation and ubiquitylation that promotes targeted degradation of UBE2F. *Mol Cell.* (2018) 70:1008.e–24.e. 10.1016/j.molcel.2018.06.002 29932898 PMC6021141

[38] Gao F Fan Y Zhou B Guo W Jiang X Shi J et al. The functions and properties of cullin-5, a potential therapeutic target for cancers. *Am J Transl Res.* (2020) 12:618–32. 10.62347/uymp7222 32194910 PMC7061844

[39] Wang L Huang J Zhang R Zhang M Guo Y Liu Y et al. Cullin 5 aggravates hypoxic pulmonary hypertension by activating TRAF6/NF-κB/HIF-1α/VEGF. *iScience.* (2023) 26:108199. 10.1016/j.isci.2023.108199 37965157 PMC10641258

[40] Chou MC Wang YH Chen FY Kung CY Wu KP Kuo JC et al. PAICS ubiquitination recruits UBAP2 to trigger phase separation for purinosome assembly. *Mol Cell.* (2023) 83:4123.e–40.e. 10.1016/j.molcel.2023.09.028 37848033

[41] Zhang S Sun Y. Cullin RING ligase 5 (CRL-5): neddylation activation and biological functions. *Adv Exp Med Biol.* (2020) 1217:261–83. 10.1007/978-981-15-1025-0_16 31898233

[42] Samant RS Clarke PA Workman P. E3 ubiquitin ligase Cullin-5 modulates multiple molecular and cellular responses to heat shock protein 90 inhibition in human cancer cells. *Proc Natl Acad Sci U S A.* (2014) 111:6834–9. 10.1073/pnas.1322412111 24760825 PMC4020071

[43] Babon JJ Sabo JK Zhang JG Nicola NA Norton RS. The SOCS box encodes a hierarchy of affinities for Cullin5: implications for ubiquitin ligase formation and cytokine signalling suppression. *J Mol Biol.* (2009) 387:162–74. 10.1016/j.jmb.2009.01.024 19385048 PMC2720833

[44] Cui B Gong L Chen M Zhang Y Yuan H Qin J et al. CUL5-SOCS6 complex regulates mTORC2 function by targeting Sin1 for degradation. *Cell Discov.* (2019) 5:52. 10.1038/s41421-019-0118-6 31798957 PMC6868212

[45] Ramachandran S Makukhin N Haubrich K Nagala M Forrester B Lynch DM et al. Structure-based design of a phosphotyrosine-masked covalent ligand targeting the E3 ligase SOCS2. *Nat Commun.* (2023) 14:6345. 10.1038/s41467-023-41894-3 37816714 PMC10564737

[46] Zhao G Gong L Su D Jin Y Guo C Yue M et al. Cullin5 deficiency promotes small-cell lung cancer metastasis by stabilizing integrin β1. *J Clin Invest.* (2019) 129:972–87. 10.1172/jci122779 30688657 PMC6391098

[47] Reyes RV Hino K Canales CP Dickson EJ La Torre A Simó S. The E3 ubiquitin ligase CRL5 regulates dentate gyrus morphogenesis, adult neurogenesis, and animal behavior. *Front Neurosci.* (2022) 16:908719. 10.3389/fnins.2022.908719 35801174 PMC9253586

[48] Simó S Cooper JA. Rbx2 regulates neuronal migration through different cullin 5-RING ligase adaptors. *Dev Cell.* (2013) 27:399–411. 10.1016/j.devcel.2013.09.022 24210661 PMC3851519

[49] Samelson AJ Ariqat N McKetney J Rohanitazangi G Bravo CP Bose R et al. CRISPR screens in iPSC-derived neurons reveal principles of tau proteostasis. *bioRxiv.* (2024). 10.1101/2023.06.16.545386 41610849 PMC12978015

[50] Querido E Blanchette P Yan Q Kamura T Morrison M Boivin D et al. Degradation of p53 by adenovirus E4orf6 and E1B55K proteins occurs via a novel mechanism involving a Cullin-containing complex. *Genes Dev.* (2001) 15:3104–17. 10.1101/gad.926401 11731475 PMC312842

[51] Cai QL Knight JS Verma SC Zald P Robertson ES. EC5S ubiquitin complex is recruited by KSHV latent antigen LANA for degradation of the VHL and p53 tumor suppressors. *PLoS Pathog.* (2006) 2:e116. 10.1371/journal.ppat.0020116 17069461 PMC1626105

[52] Sato Y Shirata N Kudoh A Iwahori S Nakayama S Murata T et al. Expression of Epstein-Barr virus BZLF1 immediate-early protein induces p53 degradation independent of MDM2, leading to repression of p53-mediated transcription. *Virology.* (2009) 388:204–11. 10.1016/j.virol.2009.03.017 19375142

[53] Sato Y Kamura T Shirata N Murata T Kudoh A Iwahori S et al. Degradation of phosphorylated p53 by viral protein-ECS E3 ligase complex. *PLoS Pathog.* (2009) 5:e1000530. 10.1371/journal.ppat.1000530 19649319 PMC2712087

[54] Levine AJ. The common mechanisms of transformation by the small DNA tumor viruses: the inactivation of tumor suppressor gene products: p53. *Virology.* (2009) 384:285–93. 10.1016/j.virol.2008.09.034 19081592

[55] Yang H Wang H Ren J Chen Q Chen ZJ. cGAS is essential for cellular senescence. *Proc Natl Acad Sci.* (2017) 114:E4612–20. 10.1073/pnas.1705499114 28533362 PMC5468617

[56] Korneenko TV Pestov NB Nevzorov IA Daks AA Trachuk KN Solopova ON et al. At the crossroads of the cGAS-cGAMP-STING pathway and the DNA damage response: implications for cancer progression and treatment. *Pharmaceuticals (Basel).* (2023) 16:1675. 10.3390/ph16121675 38139802 PMC10747911

[57] Xu P Liu Y Liu C Guey B Li L Melenec P et al. The CRL5-SPSB3 ubiquitin ligase targets nuclear cGAS for degradation. *Nature.* (2024) 627:873–9. 10.1038/s41586-024-07112-w 38418882 PMC10972748

[58] Reiner DJ Lundquist EA. Small GTPases. *WormBook.* (2018) 2018:1–65. 10.1895/wormbook.1.67.2 27218782 PMC6369420

[59] Neumann AJ Prekeris R. A Rab-bit hole: Rab40 GTPases as new regulators of the actin cytoskeleton and cell migration. *Front Cell Dev Biol.* (2023) 11:1268922. 10.3389/fcell.2023.1268922 37736498 PMC10509765

[60] Duncan ED Han KJ Trout MA Prekeris R. Ubiquitylation by Rab40b/Cul5 regulates Rap2 localization and activity during cell migration. *J Cell Biol.* (2022) 221:e202107114. 10.1083/jcb.202107114 35293963 PMC8931537

[61] Sampath R Vaeth K Mikalayeva V Skeberdis VA Prekeris R Han KJ. Rab40 GTPases regulateAMBRA1-mediated transcription and cell migration. *J Cell Sci.* (2025) 138. 10.1101/2024.11.07.622540 40110710 PMC12045048

[62] Duncan ED Lencer E Linklater E Prekeris R. Methods to study the unique SOCS box domain of the Rab40 small GTPase subfamily. *Methods Mol Biol.* (2021) 2293:163–79. 10.1007/978-1-0716-1346-7_11 34453716 PMC8455146

[63] Han KJ Mikalayeva V Gerber SA Kettenbach AN Skeberdis VA Prekeris R. Rab40c regulates focal adhesions and PP6 activity by controlling ANKRD28 ubiquitylation. *Life Sci Alliance.* (2022) 5:e202101346. 10.26508/lsa.202101346 35512830 PMC9070665

[64] Han JS Hino K Li W Reyes RV Canales CP Miltner AM et al. CRL5-dependent regulation of the small GTPases ARL4C and ARF6 controls hippocampal morphogenesis. *Proc Natl Acad Sci U S A.* (2020) 117:23073–84. 10.1073/pnas.2002749117 32873638 PMC7502717

[65] Feng L Allen NS Simo S Cooper JA. Cullin 5 regulates Dab1 protein levels and neuron positioning during cortical development. *Genes Dev.* (2007) 21:2717–30. 10.1101/gad.1604207 17974915 PMC2045127

[66] Zhou L Jiang Y Luo Q Li L Jia L. Neddylation: a novel modulator of the tumor microenvironment. *Mol Cancer.* (2019) 18:77. 10.1186/s12943-019-0979-1 30943988 PMC6446326

[67] Wu D Sun Y. Neddylation-CRLs regulate the functions of Treg immune cells. *Bioessays.* (2023) 45:e2200222. 10.1002/bies.202200222 36709423

[68] Huang DT Ayrault O Hunt HW Taherbhoy AM Duda DM Scott DC et al. E2-RING expansion of the NEDD8 cascade confers specificity to cullin modification. *Mol Cell.* (2009) 33:483–95. 10.2210/pdb3fn1/pdb 19250909 PMC2725360

[69] Zhou H Lu J Liu L Bernard D Yang CY Fernandez-Salas E et al. A potent small-molecule inhibitor of the DCN1-UBC12 interaction that selectively blocks cullin 3 neddylation. *Nat Commun.* (2017) 8:1150. 10.1038/s41467-017-01243-7 29074978 PMC5658359

[70] Zhou L Zhang W Sun Y Jia L. Protein neddylation and its alterations in human cancers for targeted therapy. *Cell Signal.* (2018) 44:92–102. 10.1016/j.cellsig.2018.01.009 29331584 PMC5829022

[71] Xiong L Liu HS Zhou C Yang X Huang L Jie HQ et al. A novel protein encoded by circINSIG1 reprograms cholesterol metabolism by promoting the ubiquitin-dependent degradation of INSIG1 in colorectal cancer. *Mol Cancer.* (2023) 22:72. 10.1186/s12943-023-01773-3 37087475 PMC10122405

[72] Zhang H Xue K Li W Yang X Gou Y Su X et al. Cullin5 drives experimental asthma exacerbations by modulating alveolar macrophage antiviral immunity. *Nat Commun.* (2024) 15:252. 10.1038/s41467-023-44168-0 38177117 PMC10766641

[73] Turek I Tischer N Lassig R Trujillo M. Multi-tiered pairing selectivity between E2 ubiquitin-conjugating enzymes and E3 ligases. *J Biol Chem.* (2018) 293:16324–36. 10.1074/jbc.ra118.004226 30185618 PMC6200922

[74] Ye Y Rape M. Building ubiquitin chains: E2 enzymes at work. *Nat Rev Mol Cell Biol.* (2009) 10:755–64. 10.1038/nrm2780 19851334 PMC3107738

[75] Draczkowski P Chen SN Chen T Wang YS Shih HA Huang JYC et al. Structural basis of K11/K48-branched ubiquitin chain recognition by the human 26S proteasome. *Nat Commun.* (2025) 16:9094. 10.1038/s41467-025-64719-x 41093839 PMC12528685

[76] Harper JW Schulman BA. Cullin-RING ubiquitin ligase regulatory circuits: a quarter century beyond the F-box hypothesis. *Annu Rev Biochem.* (2021) 90:403–29. 10.1146/annurev-biochem-090120-013613 33823649 PMC8217159

[77] Henneberg LT Singh J Duda DM Baek K Yanishevski D Murray PJ et al. Activity-based profiling of cullin-RING E3 networks by conformation-specific probes. *Nat Chem Biol.* (2023) 19:1513–23. 10.1038/s41589-023-01392-5 37653169 PMC10667097

[78] Huang T Li J Liu X Shi B Li S An HX. An integrative pan-cancer analysis revealing the difference in small ring finger family of SCF E3 ubiquitin ligases. *Front Immunol.* (2022) 13:968777. 10.3389/fimmu.2022.968777 36059474 PMC9434121

[79] Dobransky A Root M Hafner N Marcum M Sharifi HJ. CRL4-DCAF1 ubiquitin ligase dependent functions of HIV viral protein R and viral protein X. *Viruses.* (2024) 16:1313. 10.3390/v16081313 39205287 PMC11360348

[80] Diaz S Wang K Sjögren B Liu X. Roles of cullin-RING ubiquitin ligases in cardiovascular diseases. *Biomolecules.* (2022) 12:416. 10.3390/biom12030416 35327608 PMC8946067

[81] Kauppi M Hyland CD Viney EM White CA de Graaf CA Welch AE et al. Cullin-5 controls the number of megakaryocyte-committed stem cells to prevent thrombocytosis in mice. *Blood.* (2025) 145:1034–46. 10.1182/blood.2024025406 39791603 PMC11923429

[82] Onel M Sumbul F Liu J Nussinov R Haliloglu T. Cullin neddylation may allosterically tune polyubiquitin chain length and topology. *Biochem J.* (2017) 474:781–95. 10.1042/bcj20160748 28082425 PMC7900908

[83] Kostrhon S Prabu JR Baek K Horn-Ghetko D von Gronau S Klügel M et al. CUL5-ARIH2 E3-E3 ubiquitin ligase structure reveals cullin-specific NEDD8 activation. *Nat Chem Biol.* (2021) 17:1075–83. 10.1038/s41589-021-00858-8 34518685 PMC8460447

[84] Vava A Paccez JD Wang Y Gu X Bhasin MK Myers M et al. DCUN1D1 is an essential regulator of prostate cancer proliferation and tumour growth that acts through neddylation of cullin 1, 3, 4A and 5 and deregulation of Wnt/catenin pathway. *Cells.* (2023) 12:1973. 10.3390/cells12151973 37566052 PMC10417424

[85] Kim AY Bommeljé CC Lee BE Yonekawa Y Choi L Morris LG et al. SCCRO (DCUN1D1) is an essential component of the E3 complex for neddylation. *J Biol Chem.* (2008) 283:33211–20. 10.1074/jbc.m804440200 18826954 PMC2586271

[86] Paccez JD Foret CLM de Vasconcellos JF Donaldson L Zerbini LF. DCUN1D1 and neddylation: potential targets for cancer therapy. *Biochim Biophys Acta Mol Basis Dis.* (2024) 1870:167308. 10.1016/j.bbadis.2024.167308 38885797

[87] Chang Y Chen Q Li H Xu J Tan M Xiong X et al. The UBE2F-CRL5(ASB11)-DIRAS2 axis is an oncogene and tumor suppressor cascade in pancreatic cancer cells. *Dev Cell.* (2024) 59:1317.e–32.e. 10.1016/j.devcel.2024.03.018 38574733

[88] Sundquist SP Lee SE Burnatowska-Hledin M. The regulation of cellular proliferation by VACM-1/CUL5 is dependent on its posttranslational modifications by NEDD8. *The FASEB Journal.* (2019) 33:421–61. 10.1096/fasebj.2019.33.1_supplement.461.21

[89] Morsi RZ Hage-Sleiman R Kobeissy H Dbaibo G. Noxa: role in cancer pathogenesis and treatment. *Curr Cancer Drug Targets.* (2018) 18:914–28. 10.2174/1568009618666180308105048 29521234

[90] Xu S Ma Y Tong Q Yang J Liu J Wang Y et al. Cullin-5 neddylation-mediated NOXA degradation is enhanced by PRDX1 oligomers in colorectal cancer. *Cell Death Dis.* (2021) 12:265. 10.1038/s41419-021-03656-1 33712558 PMC7954848

[91] Zhou W Xu J Li H Xu M Chen ZJ Wei W et al. Neddylation E2 UBE2F promotes the survival of lung cancer cells by activating CRL5 to degrade NOXA via the K11 linkage. *Clin Cancer Res.* (2017) 23:1104–16. 10.1158/1078-0432.ccr-16-1585 27591266 PMC5315595

[92] Xu T Ma Q Li Y Yu Q Pan P Zheng Y et al. A small molecule inhibitor of the UBE2F-CRL5 axis induces apoptosis and radiosensitization in lung cancer. *Signal Transduct Target Ther.* (2022) 7:354. 10.1038/s41392-022-01182-w 36253371 PMC9576757

[93] Ma T Song Q Cheng B Guo E Wang X Li M et al. Proapoptotic effect of WS-299 induced by NOXA accumulation and NRF2-counterbalanced oxidative stress damage through targeting RBX1-UBE2M interaction in gastric cancers. *Bioorg Chem.* (2024) 144:107142. 10.1016/j.bioorg.2024.107142 38280358

[94] Tang H Pang X Li S Tang L. The double-edged effects of MLN4924: rethinking anti-cancer drugs targeting the neddylation pathway. *Biomolecules.* (2024) 14:738. 10.3390/biom14070738 39062453 PMC11274557

[95] Zhang J Bu X Wang H Zhu Y Geng Y Nihira NT et al. Cyclin D-CDK4 kinase destabilizes PD-L1 via cullin 3-SPOP to control cancer immune surveillance. *Nature.* (2018) 553:91–5. 10.1038/nature25015 29160310 PMC5754234

[96] Emanuele MJ Elia AE Xu Q Thoma CR Izhar L Leng Y et al. Global identification of modular cullin-RING ligase substrates. *Cell.* (2011) 147:459–74. 10.1016/j.cell.2011.09.019 21963094 PMC3226719

[97] Lewno MT Cui T Wang X. Cullin deneddylation suppresses the necroptotic pathway in cardiomyocytes. *Front Physiol.* (2021) 12:690423. 10.3389/fphys.2021.690423 34262479 PMC8273387

[98] Cui D Xiong X Zhao Y. Cullin-RING ligases in regulation of autophagy. *Cell Div.* (2016) 11:8. 10.1186/s13008-016-0022-5 27293474 PMC4902950

[99] Cope GA Suh GS Aravind L Schwarz SE Zipursky SL Koonin EV et al. Role of predicted metalloprotease motif of Jab1/Csn5 in cleavage of Nedd8 from Cul1. *Science.* (2002) 298:608–11. 10.1126/science.1075901 12183637

[100] Kim YM Kim HJ Kim DK Jung DH Cho HJ Kim S et al. Differential dynamics of cullin deneddylation via COP9 signalosome subunit 5 interaction. *Biochem Biophys Res Commun.* (2022) 637:341–7. 10.1016/j.bbrc.2022.11.045 36423380

[101] Zhang J Zhao R Yu C Bryant CLN Wu K Liu Z et al. IKK-mediated regulation of the COP9 signalosome via phosphorylation of CSN5. *J Proteome Res.* (2020) 19:1119–30. 10.1021/acs.jproteome.9b00626 31950832 PMC7299130

[102] Huo A Xiong X. PAICS as a potential target for cancer therapy linking purine biosynthesis to cancer progression. *Life Sci.* (2023) 331:122070. 10.1016/j.lfs.2023.122070 37673296

[103] Zhou W Yao Y Scott AJ Wilder-Romans K Dresser JJ Werner CK et al. Purine metabolism regulates DNA repair and therapy resistance in glioblastoma. *Nat Commun.* (2020) 11:3811. 10.1101/2020.03.26.010140 32732914 PMC7393131

[104] Ali ES Sahu U Villa E O’Hara BP Gao P Beaudet C et al. ERK2 phosphorylates PFAS to mediate posttranslational control of de novo purine synthesis. *Mol Cell.* (2020) 78:1178.e–91.e. 10.1016/j.molcel.2020.05.001 32485148 PMC7306006

[105] Biebl MM Buchner J. Structure, function, and regulation of the Hsp90 machinery. *Cold Spring Harb Perspect Biol.* (2019) 11:a034017. 10.1101/cshperspect.a034017 30745292 PMC6719599

[106] Bagatell R Whitesell L. Altered Hsp90 function in cancer: a unique therapeutic opportunity. *Mol Cancer Ther.* (2004) 3:1021–30. 10.1158/1535-7163.1021.3.8 15299085

[107] Imai T Kato Y Kajiwara C Mizukami S Ishige I Ichiyanagi T et al. Heat shock protein 90 (HSP90) contributes to cytosolic translocation of extracellular antigen for cross-presentation by dendritic cells. *Proc Natl Acad Sci U S A.* (2011) 108:16363–8. 10.1073/pnas.1108372108 21930907 PMC3182735

[108] Ducellier S Demeules M Letribot B Gaetani M Michaudel C Sokol H et al. Dual molecule targeting HDAC6 leads to intratumoral CD4+ cytotoxic lymphocytes recruitment through MHC-II upregulation on lung cancer cells. *J Immunother Cancer.* (2024) 12:e007588. 10.1136/jitc-2023-007588 38609101 PMC11015306

[109] Ehrlich ES Wang T Luo K Xiao Z Niewiadomska AM Martinez T et al. Regulation of Hsp90 client proteins by a Cullin5-RING E3 ubiquitin ligase. *Proc Natl Acad Sci U S A.* (2009) 106:20330–5. 10.1073/pnas.0810571106 19933325 PMC2787120

[110] Zheng-Lin B Graham RP Bekaii-Saab TS. Targeting ERBB2/HER2 genetic alterations: an expanding therapeutic opportunity in gastrointestinal cancers. *Chin Clin Oncol.* (2023) 12:55. 10.21037/cco-23-72 37964543

[111] Talaei S Mellatyar H Asadi A Akbarzadeh A Sheervalilou R Zarghami N. Spotlight on 17-AAG as an Hsp90 inhibitor for molecular targeted cancer treatment. *Chem Biol Drug Des.* (2019) 93:760–86. 10.1111/cbdd.13486 30697932

[112] Zhang Z Li Y Zhang R Yu X. Total synthesis of geldanamycin. *J Org Chem.* (2021) 86:15063–75. 10.1021/acs.joc.1c01582 34657428

[113] Cui D Shao S Qu R Chen X Jiang S Wang L et al. The FBXW7-RPAP2 axis controls the growth of hepatocellular carcinoma cells and determines the fate of liver cell differentiation. *Adv Sci (Weinh).* (2025) 12:e2404718. 10.1002/advs.202404718 39932049 PMC11967794

[114] Okumura F Fujiki Y Oki N Osaki K Nishikimi A Fukui Y et al. Cul5-type ubiquitin ligase KLHDC1 contributes to the elimination of truncated SELENOS produced by failed UGA/sec decoding. *iScience.* (2020) 23:100970. 10.1016/j.isci.2020.100970 32200094 PMC7090344

[115] Angelone T Rocca C Lionetti V Penna C Pagliaro P. Expanding the frontiers of guardian antioxidant selenoproteins in cardiovascular pathophysiology. *Antioxid Redox Signal.* (2024) 40:369–432. 10.1089/ars.2023.0285 38299513

[116] Petroski MD Deshaies RJ. Function and regulation of cullin-RING ubiquitin ligases. *Nat Rev Mol Cell Biol.* (2005) 6:9–20. 10.1038/nrm1547 15688063

[117] Liau NPD Laktyushin A Lucet IS Murphy JM Yao S Whitlock E et al. The molecular basis of JAK/STAT inhibition by SOCS1. *Nat Commun.* (2018) 9:1558. 10.1038/s41467-018-04013-1 29674694 PMC5908791

[118] Wu W Sun XH. A mechanism underlying NOTCH-induced and ubiquitin-mediated JAK3 degradation. *J Biol Chem.* (2011) 286:41153–62. 10.1074/jbc.m111.273755 21969365 PMC3308829

[119] Nie L Zhao Y Wu W Yang YZ Wang HC Sun XH. Notch-induced Asb2 expression promotes protein ubiquitination by forming non-canonical E3 ligase complexes. *Cell Res.* (2011) 21:754–69. 10.1038/cr.2010.165 21119685 PMC3085721

[120] Georgana I Maluquer de Motes C. Cullin-5 adaptor SPSB1 controls NF-κB activation downstream of multiple signaling pathways. *Front Immunol.* (2019) 10:3121. 10.3389/fimmu.2019.03121 32038638 PMC6985365

[121] Zhu Z Sun L Hao R Jiang H Qian F Ye RD. Nedd8 modification of Cullin-5 regulates lipopolysaccharide-induced acute lung injury. *Am J Physiol Lung Cell Mol Physiol.* (2017) 313:L104–14. 10.1152/ajplung.00410.2016 28522566

[122] Zhu Z Wang L Hao R Zhao B Sun L Ye RD. Cutting edge: a cullin-5-TRAF6 interaction promotes TRAF6 polyubiquitination and lipopolysaccharide signaling. *J Immunol.* (2016) 197:21–6. 10.4049/jimmunol.1600447 27233966

[123] Guangwei Z Zhibin C Qin W Chunlin L Penghang L Ruofan H et al. TRAF6 regulates the signaling pathway influencing colorectal cancer function through ubiquitination mechanisms. *Cancer Sci.* (2022) 113:1393–405. 10.1111/cas.15302 35179811 PMC8990288

[124] Szwed A Kim E Jacinto E. Regulation and metabolic functions of mTORC1 and mTORC2. *Physiol Rev.* (2021) 101:1371–426. 10.1152/physrev.00026.2020 33599151 PMC8424549

[125] Xu M Zhou Y Fan S Zhang M Gao X. Cul5 mediates taurine-stimulated mTOR mRNA expression and proliferation of mouse mammary epithelial cells. *Amino Acids.* (2023) 55:243–52. 10.1007/s00726-022-03222-9 36449095

[126] Ma N He F Kawanokuchi J Wang G Yamashita T. Taurine and its anticancer functions: in vivo and in vitro study. *Adv Exp Med Biol.* (2022) 1370:121–8. 10.1007/978-3-030-93337-1_11 35882787

[127] Antonioli M Albiero F Nazio F Vescovo T Perdomo AB Corazzari M et al. AMBRA1 interplay with cullin E3 ubiquitin ligases regulates autophagy dynamics. *Dev Cell.* (2014) 31:734–46. 10.1016/j.devcel.2014.11.013 25499913

[128] King KE Losier TT Russell RC. Regulation of autophagy enzymes by nutrient signaling. *Trends Biochem Sci.* (2021) 46:687–700. 10.1016/j.tibs.2021.01.006 33593593

[129] Gong L Wang K Wang M Hu R Li H Gao D et al. CUL5-ASB6 complex promotes p62/SQSTM1 ubiquitination and degradation to regulate cell proliferation and autophagy. *Front Cell Dev Biol.* (2021) 9:684885. 10.3389/fcell.2021.684885 34164402 PMC8215545

[130] Gao X Yu Y Sun J Zhao H You Y Shi X et al. CUL5 E3 ubiquitin ligase regulates the evasion of bladder cancer cells to CD8+ T cell-mediated killing by inhibiting autophagy. *PLoS Biol.* (2026) 24:e3003647. 10.1371/journal.pbio.3003647 41662369 PMC12900434

[131] Campos Y Rodriguez-Enriquez R Palacios G Van de Vlekkert D Qiu X Weesner J et al. Mitochondrial proteostasis mediated by CRL5 (Ozz) and Alix maintains skeletal muscle function. *bioRxiv.* (2023). 10.1101/2023.07.11.548601 37503076 PMC10369959

[132] Wang W Li E Zou J Qu C Ayala J Wen Y et al. The ubiquitin ligase RBX2/SAG regulates mitochondrial ubiquitination and mitophagy. *bioRxiv.* (2024). 10.1101/2024.02.24.581168 38873758 PMC11264309

[133] Huang B Song BL Xu C. Cholesterol metabolism in cancer: mechanisms and therapeutic opportunities. *Nat Metab.* (2020) 2:132–41. 10.1038/s42255-020-0174-0 32694690

[134] Chan CH Li CF Yang WL Gao Y Lee SW Feng Z et al. The Skp2-SCF E3 ligase regulates Akt ubiquitination, glycolysis, herceptin sensitivity, and tumorigenesis. *Cell.* (2012) 149:1098–111. 10.1016/j.cell.2012.02.065 22632973 PMC3586339

[135] Sudhagar S Sathya S Lakshmi BS. Rapid non-genomic signalling by 17β-oestradiol through c-Src involves mTOR-dependent expression of HIF-1α in breast cancer cells. *Br J Cancer.* (2011) 105:953–60. 10.1038/bjc.2011.349 21897387 PMC3185958

[136] Scheufele F Wolf B Kruse M Hartmann T Lempart J Mühlich S et al. Evidence for a regulatory role of Cullin-RING E3 ubiquitin ligase 7 in insulin signaling. *Cell Signal.* (2014) 26:233–9. 10.1016/j.cellsig.2013.11.005 24219910 PMC3901049

[137] D’Amato RJ Loughnan MS Flynn E Folkman J. Thalidomide is an inhibitor of angiogenesis. *Proc Natl Acad Sci U S A.* (1994) 91:4082–5. 10.1073/pnas.91.9.4082 7513432 PMC43727

[138] Kunkler B Salamango D DeBruine ZJ Ploch C Dean S Grossens D et al. CUL5 is required for thalidomide-dependent inhibition of cellular proliferation. *PLoS One.* (2018) 13:e0196760. 10.1371/journal.pone.0196760 29746508 PMC5944951

[139] Okumura F Joo-Okumura A Nakatsukasa K Kamura T. The role of cullin 5-containing ubiquitin ligases. *Cell Div.* (2016) 11:1. 10.1186/s13008-016-0016-3 27030794 PMC4812663

[140] Shi Z Yao J Ma X Xu D Ming G. CUL5-mediated visfatin (NAMPT) degradation blocks endothelial proliferation and angiogenesis via the MAPK/PI3K-AKT signaling. *J Cardiovasc Pharmacol.* (2021) 78:891–9. 10.1097/fjc.0000000000001146 34596622

[141] Buchwalter A Van Dort C Schultz S Smith R Le IP Abbott JL et al. Expression of VACM-1/cul5 mutant in endothelial cells induces MAPK phosphorylation and maspin degradation and converts cells to the angiogenic phenotype. *Microvasc Res.* (2008) 75:155–68. 10.1016/j.mvr.2007.08.004 17950367

[142] Johnson AE Le IP Andresen BT Stodola J Dewey GL Dean SB et al. VACM-1/cul5 expression in vascular tissue in vivo is induced by water deprivation and its expression in vitro regulates aquaporin-1 concentrations. *Cell Tissue Res.* (2012) 349:527–39. 10.1007/s00441-012-1419-3 22581383

[143] Murali SK McCormick JA Fenton RA. Regulation of the water channel aquaporin-2 by cullin E3 ubiquitin ligases. *Am J Physiol Renal Physiol.* (2024) 326:F814–26. 10.1152/ajprenal.00049.2024 38545647 PMC11381000

[144] Le IP Schultz S Andresen BT Dewey GL Zhao P Listenberger L et al. Aquaporin-2 levels in vitro and in vivo are regulated by VACM-1, a cul 5 gene. *Cell Physiol Biochem.* (2012) 30:1148–58. 10.1159/000343305 23171819

[145] Chen S Shao F Zeng J Guo S Wang L Sun H et al. Cullin-5 deficiency orchestrates the tumor microenvironment to promote mammary tumor development through CREB1-CCL2 signaling. *Sci Adv.* (2023) 9:eabq1395. 10.1126/sciadv.abq1395 36662868 PMC9858512

[146] Warmack RA Pang EZ Peluso E Lowenson JD Ong JY Torres JZ et al. Human protein-l-isoaspartate O-methyltransferase domain-containing protein 1 (PCMTD1) associates with cullin-RING ligase proteins. *Biochemistry.* (2022) 61:879–94. 10.1021/acs.biochem.2c00130 35486881 PMC9875861

[147] Kumar B Field NS Kim DD Dar AA Chen Y Suresh A et al. The ubiquitin ligase Cul5 regulates CD4(+) T cell fate choice and allergic inflammation. *Nat Commun.* (2022) 13:2786. 10.1038/s41467-022-30437-x 35589717 PMC9120070

[148] Shang Q Yu X Sun Q Li H Sun C Liu L. Polysaccharides regulate Th1/Th2 balance: a new strategy for tumor immunotherapy. *Biomed Pharmacother.* (2024) 170:115976. 10.1016/j.biopha.2023.115976 38043444

[149] Anandasabapathy N Ford GS Bloom D Holness C Paragas V Seroogy C et al. GRAIL: an E3 ubiquitin ligase that inhibits cytokine gene transcription is expressed in anergic CD4+ T cells. *Immunity.* (2003) 18:535–47. 10.1016/s1074-7613(03)00084-0 12705856

[150] Fathman CG Yip L Gómez-Martín D Yu M Seroogy CM Hurt CR et al. How GRAIL controls Treg function to maintain self-tolerance. *Front Immunol.* (2022) 13:1046631. 10.3389/fimmu.2022.1046631 36569931 PMC9773990

[151] Ma Y Liao J Cheng H Yang Q Yang H. Advanced gene therapy system for the treatment of solid tumour: a review. *Mater Today Bio.* (2024) 27:101138. 10.1016/j.mtbio.2024.101138 39027677 PMC11255123

[152] Zhou Y Wang X Tian X Zhang D Cui H Du W et al. Stealth missiles with precision guidance: a novel multifunctional nano-drug delivery system based on biomimetic cell membrane coating technology. *Mater Today Bio.* (2025) 33:101922. 10.1016/j.mtbio.2025.101922 40528836 PMC12173624

[153] Canè S Ugel S Trovato R Marigo I De Sanctis F Sartoris S et al. The endless saga of monocyte diversity. *Front Immunol.* (2019) 10:1786. 10.3389/fimmu.2019.01786 31447834 PMC6691342

[154] Hao Q Vadgama JV Wang P. CCL2/CCR2 signaling in cancer pathogenesis. *Cell Commun Signal.* (2020) 18:82. 10.1186/s12964-020-00589-8 32471499 PMC7257158

[155] Alard JE Ortega-Gomez A Wichapong K Bongiovanni D Horckmans M Megens RT et al. Recruitment of classical monocytes can be inhibited by disturbing heteromers of neutrophil HNP1 and platelet CCL5. *Sci Transl Med.* (2015) 7:317ra196. 10.1126/scitranslmed.aad5330 26659570

[156] Giladi A Wagner LK Li H Dörr D Medaglia C Paul F et al. Cxcl10(+) monocytes define a pathogenic subset in the central nervous system during autoimmune neuroinflammation. *Nat Immunol.* (2020) 21:525–34. 10.1038/s41590-020-0661-1 32313246

[157] Metzemaekers M Mortier A Vacchini A Boff D Yu K Janssens R et al. Endogenous modification of the chemoattractant CXCL5 alters receptor usage and enhances its activity toward neutrophils and monocytes. *Sci Signal.* (2021) 14:eaax3053. 10.1126/scisignal.aax3053 33688078

[158] Balamayooran G Batra S Cai S Mei J Worthen GS Penn AL et al. Role of CXCL5 in leukocyte recruitment to the lungs during secondhand smoke exposure. *Am J Respir Cell Mol Biol.* (2012) 47:104–11. 10.1165/rcmb.2011-0260oc 22362385 PMC3402800

[159] Pietras EM. Cullin’ the herd: Cul5 limits megakaryocyte-biased HSCs. *Blood.* (2025) 145:996–8. 10.1182/blood.2024027680 40048228

[160] Tomishima SA Kim DD Porter N Guha I Dar AA Ortega-Burgos Y et al. The E3 ubiquitin ligase Cul5 regulates hematopoietic stem cell function for steady-state hematopoiesis in mice. *J Clin Invest.* (2025) 135:e180913. 10.1172/jci180913 40569692 PMC12404750

[161] Teckchandani A Laszlo GS Simó S Shah K Pilling C Strait AA et al. Cullin 5 destabilizes Cas to inhibit Src-dependent cell transformation. *J Cell Sci.* (2014) 127:509–20. 10.1242/jcs.127829 24284072 PMC4007763

[162] Ferragut Cardoso AP Banerjee M Nail AN Lykoudi A States JC. miRNA dysregulation is an emerging modulator of genomic instability. *Semin Cancer Biol.* (2021) 76:120–31. 10.1016/j.semcancer.2021.05.004 33979676 PMC8576067

[163] Zhu Y Li L Hou D Ouyang Y Guo X Wang Y et al. MicroRNA-19a regulates the proliferation, migration and invasion of human gastric cancer cells by targeting CUL5. *Arch Biochem Biophys.* (2019) 662:93–100. 10.1016/j.abb.2018.11.023 30521783

[164] Wang Y Hu J-S Qi G-Y Wang S Gao J. miR-19a promotes the metastasis and EMT through CUL5 in prostate cancer cell line PC3. *J BUON.* (2020) 25:2028–35. 33099949

[165] Ma C Qi Y Shao L Liu M Li X Tang H. Downregulation of miR-7 upregulates Cullin 5 (CUL5) to facilitate G1/S transition in human hepatocellular carcinoma cells. *IUBMB Life.* (2013) 65:1026–34. 10.1002/iub.1231 24339204

[166] Nakashima S Jinnin M Ide M Kajihara I Igata T Harada M et al. A potential significance of circ_0024169 down regulation in angiosarcoma tissue. *Intractable Rare Dis Res.* (2019) 8:129–33. 10.5582/irdr.2019.01034 31218163 PMC6557243

[167] Zheng Q Bao C Guo W Li S Chen J Chen B et al. Circular RNA profiling reveals an abundant circHIPK3 that regulates cell growth by sponging multiple miRNAs. *Nat Commun.* (2016) 7:11215. 10.1038/ncomms11215 27050392 PMC4823868

[168] Bachmayr-Heyda A Reiner AT Auer K Sukhbaatar N Aust S Bachleitner-Hofmann T et al. Correlation of circular RNA abundance with proliferation–exemplified with colorectal and ovarian cancer, idiopathic lung fibrosis, and normal human tissues. *Sci Rep.* (2015) 5:8057. 10.1038/srep08057 25624062 PMC4306919

[169] Zhang L Hua M Wang Y Sun A. Comprehensive analysis of the Cullin family of genes reveals that CUL7 and CUL9 are the significant prognostic biomarkers in colorectal cancer. *Am J Transl Res.* (2024) 16:1907–24. 10.62347/chib8915 38883340 PMC11170594

[170] Lubbers J Lewis S Harper E Hledin MP Marquez GA Johnson AE et al. Resveratrol enhances anti-proliferative effect of VACM-1/cul5 in T47D cancer cells. *Cell Biol Toxicol.* (2011) 27:95–105. 10.1007/s10565-010-9173-3 20949323

[171] Kabir S Cidado J Andersen C Dick C Lin PC Mitros T et al. The CUL5 ubiquitin ligase complex mediates resistance to CDK9 and MCL1 inhibitors in lung cancer cells. *Elife.* (2019) 8:e44288. 10.1101/518555 31294695 PMC6701926

[172] He ZX Yang WG Zengyangzong D Gao G Zhang Q Liu HM et al. Targeting cullin neddylation for cancer and fibrotic diseases. *Theranostics.* (2023) 13:5017–56. 10.7150/thno.78876 37771770 PMC10526667

[173] Zhao X Xiong J Li D Zhang Y. Clinical trials of nanoparticle-enhanced CAR-T and NK cell therapies in oncology: overcoming translational and clinical challenges - a mini review. *Front Med (Lausanne).* (2025) 12:1655693. 10.3389/fmed.2025.1655693 40904365 PMC12403998

[174] Deng X Liu X Zhao L. Multifunctional nanoplatforms for tumor microenvironment remodeling: toward precision and intelligent cancer therapy. *Mater Today Bio.* (2025) 35:102385. 10.1016/j.mtbio.2025.102385 41142435 PMC12549778

[175] Ma X Li X Luo G Jiao J. DNA-functionalized gold nanoparticles: modification, characterization, and biomedical applications. *Front Chem.* (2022) 10:1095488. 10.3389/fchem.2022.1095488 36583149 PMC9792995

[176] Rangel-López R Franco-Molina M Rodríguez-Padilla C Zárate-Triviño DG. Gold nanoparticles synthesized with triple-negative breast cancer cell lysate enhance antitumoral immunity: a novel synthesis method. *Pharmaceuticals (Basel).* (2025) 18:330. 10.3390/ph18030330 40143109 PMC11945454

[177] Alobaid MA Richards SJ Alexander MR Gibson MI Ghaemmaghami AM. Monosaccharide coating modulate the intracellular trafficking of gold nanoparticles in dendritic cells. *Mater Today Bio.* (2024) 29:101371. 10.1016/j.mtbio.2024.101371 39698001 PMC11652954

[178] Luo Q Yang J Yang M Wang Y Liu Y Liu J et al. Utilization of nanotechnology to surmount the blood-brain barrier in disorders of the central nervous system. *Mater Today Bio.* (2025) 31:101457. 10.1016/j.mtbio.2025.101457 39896289 PMC11786670

[179] Menendez JB Fusco M Piccioni F Benitez CA Rizzo MM Andreozzi P et al. Glucose-functionalized gold nanoparticles for effective photothermal therapy in lung cancer. *Sci Rep.* (2025) 15:41006. 10.1038/s41598-025-24797-9 41266406 PMC12635407

[180] Bai L Hao X Xu W Huang H Guo M Zhang Y et al. FMRP silencing via siRNA lipid nanoparticles to reprogram the tumor microenvironment and enhance anti-PD-1 efficacy in triple-negative breast cancer. *Mater Today Bio.* (2025) 33:102075. 10.1016/j.mtbio.2025.102075 40727077 PMC12303082

[181] Rurik JG Tombácz I Yadegari A Méndez Fernández PO Shewale SV Li L et al. CAR T cells produced in vivo to treat cardiac injury. *Science.* (2022) 375:91–6. 10.1126/science.abm0594 34990237 PMC9983611

[182] Zhang Y Gao Z Yang X Xu Q Lu Y. Leveraging high-throughput screening technologies in targeted mRNA delivery. *Mater Today Bio.* (2024) 26:101101. 10.1016/j.mtbio.2024.101101 38883419 PMC11176929

[183] Zeng S Tang Q Xiao M Tong X Yang T Yin D et al. Cell membrane-coated nanomaterials for cancer therapy. *Mater Today Bio.* (2023) 20:100633. 10.1016/j.mtbio.2023.100633 37128288 PMC10148189

[184] Paun RA Jurchuk S Tabrizian M. A landscape of recent advances in lipid nanoparticles and their translational potential for the treatment of solid tumors. *Bioeng Transl Med.* (2024) 9:e10601. 10.1002/btm2.10601/v2/response1 38435821 PMC10905562

[185] Peng X Wang J Deng Z Wei J Xie C Wang Y et al. NIR laser-activated phthalocyanine loaded lipid nanoparticles targeting M2 macrophage for improved photoacoustic imaging-guided photothermal therapy. *Mater Today Bio.* (2024) 28:101209. 10.1016/j.mtbio.2024.101209 39221205 PMC11364919

[186] Billingsley MM Gong N Mukalel AJ Thatte AS El-Mayta R Patel SK et al. In vivo mRNA CAR T cell engineering via targeted ionizable lipid nanoparticles with extrahepatic tropism. *Small.* (2024) 20:e2304378. 10.1002/smll.202304378 38072809

[187] Chen Q Yang Z Liu H Man J Oladejo AO Ibrahim S et al. Novel drug delivery systems: an important direction for drug innovation research and development. *Pharmaceutics.* (2024) 16:674. 10.3390/pharmaceutics16050674 38794336 PMC11124876

[188] Tatar AS Nagy-Simon T Tigu AB Tomuleasa C Boca S. Optimization of tyrosine kinase inhibitor-loaded gold nanoparticles for stimuli-triggered antileukemic drug release. *J Funct Biomater.* (2023) 14:399. 10.21203/rs.3.rs-2314501/v1 37623644 PMC10455807

[189] Nakka NMR Rachamala HK Angom RS Indla NR Dutta SK Wang E et al. Dual drug-loaded tumor-targeted polymeric nanoparticles for enhancing therapeutic response in pancreatic ductal adenocarcinoma. *Mater Today Bio.* (2024) 28:101199. 10.1016/j.mtbio.2024.101199 39205875 PMC11357805

[190] Oh H Jeong E Lee JS Kim J Lee D Kim BS et al. ROS-responsive PEGylated ferrocene polymer nanoparticles with improved stability for tumor-selective chemotherapy and imaging. *Mater Today Bio.* (2023) 22:100774. 10.1016/j.mtbio.2023.100774 37664795 PMC10468360

[191] Beach MA Nayanathara U Gao Y Zhang C Xiong Y Wang Y et al. Polymeric nanoparticles for drug delivery. *Chem Rev.* (2024) 124:5505–616. 10.1021/acs.chemrev.3c00705 38626459 PMC11086401

[192] Qian J Liu Y. Recent advances in adoptive cell therapy for cancer immunotherapy. *Front Immunol.* (2025) 16:1665488. 10.3389/fimmu.2025.1665488 41058692 PMC12497837

[193] Yoon DH Osborn MJ Tolar J Kim CJ. Incorporation of immune checkpoint blockade into chimeric antigen receptor T cells (CAR-Ts): combination or built-in CAR-T. *Int J Mol Sci.* (2018) 19:340. 10.3390/ijms19020340 29364163 PMC5855562

[194] Pan Y Yoon S Sun J Huang Z Lee C Allen M et al. Mechanogenetics for the remote and noninvasive control of cancer immunotherapy. *Proc Natl Acad Sci U S A.* (2018) 115:992–7. 10.1073/pnas.1714900115 29343642 PMC5798350

[195] Tang M Qu Y He P Yao E Guo T Yu D et al. Heat-inducible CAR-T overcomes adverse mechanical tumor microenvironment in a 3D bioprinted glioblastoma model. *Mater Today Bio.* (2024) 26:101077. 10.1016/j.mtbio.2024.101077 38765247 PMC11099333

[196] Verma A Yu C Bachl S Lopez I Schwartz M Moen E et al. Cellular behavior analysis from live-cell imaging of TCR T cell-cancer cell interactions. *bioRxiv.* (2024). 10.1101/2024.11.19.624390 39605616 PMC11601648

[197] Ryu M Kim E Kim S Kim K. Natural killer cell membrane manipulation for augmented immune synapse and anticancer efficacy. *Mater Today Bio.* (2025) 33:101965. 10.1016/j.mtbio.2025.101965 40585037 PMC12205663

[198] Mantovani A Allavena P Marchesi F Garlanda C. Macrophages as tools and targets in cancer therapy. *Nat Rev Drug Discov.* (2022) 21:799–820. 10.1038/s41573-022-00520-5 35974096 PMC9380983

[199] Ortega-Burgos Y Dar AA Tomishima SA Guha I Brien CO Porter N et al. Loss of Cullin 5 in myeloid cells protects against autoimmune neuroinflammation. *Front Immunol.* (2025) 16:1611818. 10.3389/fimmu.2025.1611818 40842987 PMC12366467

[200] Najafi S Mortezaee K. Advances in dendritic cell vaccination therapy of cancer. *Biomed Pharmacother.* (2023) 164:114954. 10.1016/j.biopha.2023.114954 37257227

[201] Galati D Zanotta S. Dendritic cell and cancer therapy. *Int J Mol Sci.* (2023) 24:4253. 10.3390/ijms24044253 36835665 PMC9968100

[202] Bano I Soomro AS Abbas SQ Ahmadi A Hassan SSU Behl T et al. Comprehensive review of biological roles and interactions of cullin-5 protein. *ACS Omega.* (2022) 7:5615–24. 10.1021/acsomega.1c06890 35224323 PMC8867543

[203] Ali A DiPersio JF. ReCARving the future: bridging CAR T-cell therapy gaps with synthetic biology, engineering, and economic insights. *Front Immunol.* (2024) 15:1432799. 10.3389/fimmu.2024.1432799 39301026 PMC11410633

